# Intermittent fasting promotes adipose thermogenesis and metabolic homeostasis via VEGF-mediated alternative activation of macrophage

**DOI:** 10.1038/cr.2017.126

**Published:** 2017-10-17

**Authors:** Kyoung-Han Kim, Yun Hye Kim, Joe Eun Son, Ju Hee Lee, Sarah Kim, Min Seon Choe, Joon Ho Moon, Jian Zhong, Kiya Fu, Florine Lenglin, Jeong-Ah Yoo, Philip J Bilan, Amira Klip, Andras Nagy, Jae-Ryong Kim, Jin Gyoon Park, Samer MI Hussein, Kyung-Oh Doh, Chi-chung Hui, Hoon-Ki Sung

**Affiliations:** 1 Developmental & Stem Cell Biology Program, The Hospital for Sick Children, Toronto, Ontario, Canada; 2 Translational Medicine Program, The Hospital for Sick Children, Toronto, Ontario M5G 1X8, Canada; 3 Department of Laboratory Medicine and Pathobiology, University of Toronto, Toronto, Ontario M5S 1A1, Canada; 4 Cell Biology Program, The Hospital for Sick Children, Toronto, Ontario, Canada; 5 Lunenfeld-Tanenbaum Research Institute, Mount Sinai Hospital, Toronto, Ontario, Canada; 6 Department of Obstetrics & Gynaecology and Institute of Medical Science, University of Toronto, Toronto, Ontario, Canada; 7 Department of Biochemistry and Molecular Biology, Smart-aging Convergence Research Center, College of Medicine, Yeungnam University, Daegu, Republic of Korea; 8 Virginia G. Piper Center for Personalized Diagnostics, Biodesign Institute, Arizona State University, Tempe, AZ, USA; 9 Centre Hospitalier Universitaire de Québec Research Center and Faculty of Medicine, Laval University, Quebec City, Quebec, Canada; 10 Department of Physiology, College of Medicine, Yeungnam University, Daegu, Republic of Korea; 11 Department of Molecular Genetics, University of Toronto, Toronto, Ontario M5S 1A1, Canada; 12 Current address: University of Ottawa Heart Institute and Department of Cellular and Molecular Medicine, Faculty of Medicine, University of Ottawa, Ottawa, Ontario, Canada

**Keywords:** intermittent fasting, thermogenesis, vascular endothelial growth factor, adipose macrophage

## Abstract

Intermittent fasting (IF), a periodic energy restriction, has been shown to provide health benefits equivalent to prolonged fasting or caloric restriction. However, our understanding of the underlying mechanisms of IF-mediated metabolic benefits is limited. Here we show that isocaloric IF improves metabolic homeostasis against diet-induced obesity and metabolic dysfunction primarily through adipose thermogenesis in mice. IF-induced metabolic benefits require fasting-mediated increases of vascular endothelial growth factor (VEGF) expression in white adipose tissue (WAT). Furthermore, periodic adipose-VEGF overexpression could recapitulate the metabolic improvement of IF in non-fasted animals. Importantly, fasting and adipose-VEGF induce alternative activation of adipose macrophage, which is critical for thermogenesis. Human adipose gene analysis further revealed a positive correlation of adipose VEGF-M2 macrophage-WAT browning axis. The present study uncovers the molecular mechanism of IF-mediated metabolic benefit and suggests that isocaloric IF can be a preventive and therapeutic approach against obesity and metabolic disorders.

## Introduction

While fat (white adipose tissue, WAT) is often associated with development of obesity and type 2 diabetes, it is essential for energy homeostasis by storing excess energy and releasing lipids in response to energy deficits^1,2^. Recent studies have discovered that WAT also contributes to whole-body metabolism by regulating thermogenic activity via the browning of WAT, which increases energy expenditure and improves insulin sensitivity^3^. In this regard, WAT browning has been suggested as a therapeutic approach for obesity and metabolic diseases. A variety of physiological stimuli (e.g., cold exposure, exercise) and signaling ligands (e.g., FGF21, BMP, VEGF) have been identified for their potential to induce WAT browning^4^. In addition, recent studies highlight the critical role of the innate immune response with alternative activation of adipose macrophage (M2 macrophage) in the regulation of metabolic function of adipose tissues, including WAT browning^5^.

We and others have previously demonstrated that vascular endothelial growth factor in adipose tissues (adipose-VEGF) plays a key role in browning of WAT and maintaining the functional integrity of adipose tissues, suggesting the therapeutic potential of VEGF in the treatment of metabolic disorders^6,7,8,9^. For example, cold acclimatization^10^, exercise^11^, and environmental stimuli^9^ promote the expression of adipose-VEGF and hence WAT browning. However, the molecular mechanism underlying VEGF-mediated WAT browning remains elusive.

Modern lifestyles favor longer periods of daily energy intake and shorter fasting periods. This erratic eating pattern is associated with metabolic disadvantages and contributes to the current global obesity and diabetes epidemic^12^. Fasting brings various positive health impacts, suggesting that modulation of fasting period can be used as a therapeutic intervention^13,14^. Intermittent fasting (IF), a periodic energy restriction method, has been shown to provide health benefits equivalent to prolonged fasting or caloric restriction (CR)^14,15,16,17,18^. The beneficial effects of IF against aging, cancer, cardiovascular diseases, and neurodegenerative diseases have been studied in both animal models and clinical settings^15,19,20,21^. However, it is unclear whether the metabolic benefits conferred by IF are primarily mediated by reduced food intake^19,22,23^ or attributed to changes in eating pattern. In particular, the 'alternate day fasting' regimen (i.e., 1 day feeding-1 day fasting, 1:1 IF), which is often used in rodent IF models, could result in underfeeding^19,24^. In addition, while WAT is one of the most critical organs for fasting physiology, it is not known whether the molecular alteration of WAT after IF is a driver or an outcome of IF-mediated metabolic benefits.

In this study, we establish a new IF regimen to investigate the impact of IF under isocaloric conditions and demonstrate that IF improves glucose homeostasis and prevents diet-induced metabolic dysfunction without caloric intake reduction. Mechanistically, we found that WAT is pivotal for mediating IF-induced metabolic benefits via browning of WAT through adipose-VEGF-mediated alternative activation of adipose macrophage (M2 macrophage). Our study unveils a novel mechanism by which IF promotes whole-body homeostasis through browning of WAT by VEGF-mediated macrophage switching.

## Results

### An IF regimen protects mice from diet-induced metabolic abnormalities

**Figure 1:**
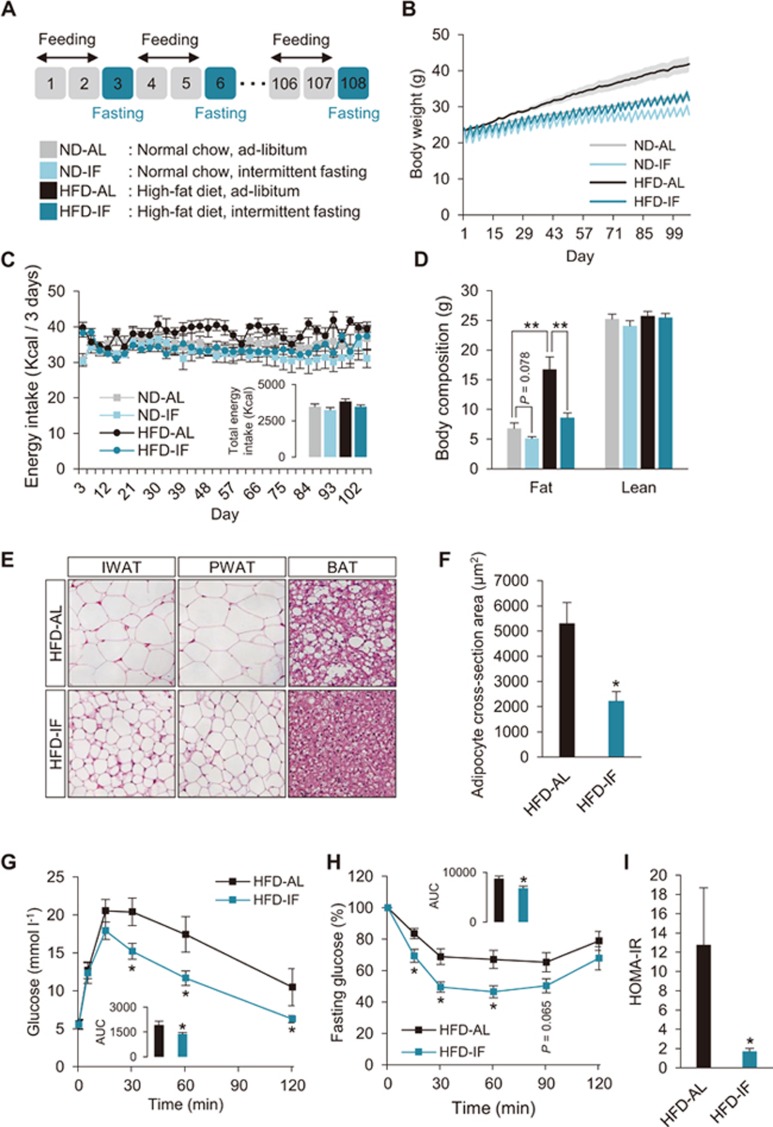
IF protects mice from diet-induced obesity and metabolic dysfunction. **(A)** Schematic illustration of the 2:1 IF regimen. **(B)** Body weight measurement during 16 weeks of IF. **(C)** Changes of energy intake during 16 weeks of IF cycles. The inset shows total energy intake during 16 weeks of IF cycles. **(D)** Body composition showing fat and lean mass. **(E)** H&E-stained sections of adipose tissues; IWAT (subcutaneous), PWAT (visceral), and BAT. **(F)** Average of cross-sectioned area of subcutaneous white adipocytes, revealing reduced white adipocyte size for HFD-IF mice compared to HFD-AL mice. **(G)** GTT in HFD-AL and HFD-IF mice. The inset shows AUC. **(H)** ITT. The inset shows AUC. **(I)** HOMA-IR. Data are expressed as mean ± SEM (ND-AL: *n* = 7; ND-IF: *n* = 8; HFD-AL: *n* = 7; and HFD-IF: *n* = 8); one or two-way ANOVA with Student-Newman-Keuls *post hoc* analysis and two-tailed unpaired Student's *t*-test; ^*^P < 0.05 and ^**^P < 0.01 vs HFD-AL. AL, *ad libitum*; AUC, area under the curve; HFD, high-fat diet; ND, normal diet.

To minimize the differences in caloric intake which may be caused by the alternate day fasting^19^, we developed a new IF regimen comprising 2 day feeding-1 day fasting periods (2:1 IF; Figure 1A). This regimen provided mice with sufficient time to compensate for the decreased body weight and for the reduced amount of food intake after 1-day fasting, to the level of non-fasted animals, enabling us to examine the effects of IF, independent of caloric intake difference (Supplementary information, Figure S1A-S1C).

Eight-week-old mice were subjected to 16 weeks of the 2:1 IF regimen on either normal chow diet (ND) or 45% high-fat diet (HFD). Compared to mice fed *ad libitum* (AL), IF mice showed lower body weight on both ND and HFD (Figure 1B; Supplementary information, Figure S1D and S1E). In further analyses, we focused mainly on IF and AL mice fed HFD (HFD-IF and HFD-AL) because they showed a marked difference in weight gain than those fed ND (Supplementary information, Figure S1F). We speculated that the reduced weight gain of IF animals might be due to the slight decrease in accumulated energy intake over 16 weeks of the diet program (Figure 1C). However, our pair-feeding experiments consistently showed that IF animals exhibited reduced weight gain, compared to mice pair-fed *ad libitum* with exactly the same amount of food as IF mice (HFD-PF; Supplementary information, Figure S2A-S2C). These data suggest that IF-mediated decrease in body weight is not primarily attributed to an energy intake difference. Body composition analysis revealed reduced fat mass without changes of lean mass in IF mice (Figure 1D and Supplementary information, Figure S2D). Consistently, HFD-IF mice exhibited a reduction in tissue weight and adipocyte size of both inguinal WAT (IWAT) and perigonadal WAT (PWAT) depots, compared to HFD-AL mice (Figure 1E and 1F; Supplementary information, Figure S1G). Lipid accumulation in brown adipose tissue (BAT) was also decreased in HFD-IF, compared to HFD-AL mice (Figure 1E). Notably, HFD-IF mice showed improved glucose homeostasis with smaller glucose excursion in glucose tolerance test (GTT), increased insulin sensitivity in insulin tolerance test (ITT), and markedly lower homeostasis model assessment-estimated insulin resistance (HOMA-IR), compared to HFD-AL or HFD-PF mice (Figure 1G-1I; Supplementary information, Figure S2E and S2F). Moreover, IF prevented HFD-induced hepatic steatosis. IF mice exhibited lower liver weight, less lipid accumulation in liver, and lower plasma alanine aminotransferase (ALT) activity (Supplementary information, Figure S1H-S1L). Together, these findings demonstrate that, in the absence of any energy intake difference, IF offers metabolic benefits against diet-induced obesity and metabolic dysfunction.

We also tested the therapeutic potential of IF on established metabolic abnormalities by performing 2:1 IF on obese mice induced by HFD for 12 weeks (post HFD; Supplementary information, Figure S3A) and were able to observe beneficial effects as early as 6 weeks of IF. This short-term 2:1 IF treatment on post HFD mice resulted in a slight reduction in body weight without changes in total caloric intake (Supplementary information, Figure S3B and S3C). Consistent with our results above, IF led to a selective decrease in fat mass, particularly in WAT mass and adipocyte size, whereas lean mass was not affected (Supplementary information, Figure S3D-S3F). Liver function parameters were also improved by 6 weeks of IF (Supplementary information, Figure S3G-S3J). Importantly, short-term IF treatment significantly improved systemic glucose homeostasis as assessed by GTT and HOMA-IR, compared to untreated mice (Supplementary information, Figure S3K and S3L). Together, these data indicate that IF is not only protective method, but also effective therapeutics against obesity.

### Transcriptome analysis of IF-treated WAT

**Figure 2:**
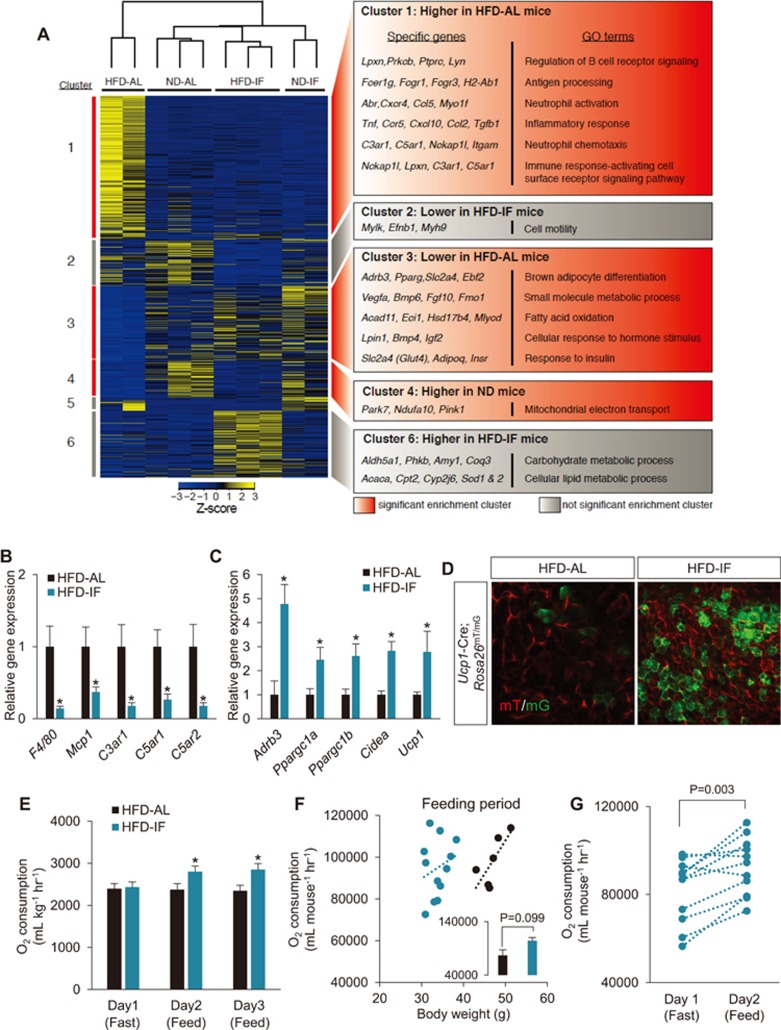
Transcriptome analysis upon IF reveals browning of WAT. **(A)** Heatmap displaying 3 644 differentially expressed genes among ND-AL, ND-IF, HFD-AL, and HFD-IF groups that were clustered into six distinct gene groups. Enriched GO terms and representative genes are shown at the right for each cluster. Significantly enriched clusters: red boxes. Not significantly enriched clusters: gray boxes. **(B)** Gene expression analysis on inflammation-related genes. **(C)** Marker gene expression of sympathetic activation (i.e., *Adrb3*) and brown/beige adipocyte (i.e., *Ppargc1a*, *Ppargc1b*, *Cidea* and *Ucp1*) (HFD, AL/IF: *n* = 6/8). **(D)** Representative images of whole-mount WAT of the brown/beige chaser (*Ucp1-Cre*;*Rosa26^mT/mG^*) mice subjected to AL and IF. Beige adipocytes are visualized by membrane-targeted GFP (mG; green). **(E)** O_2_ consumption during fasting and feeding condition. **(F)** Linear regression analysis of O_2_ consumption as a function of body weight during feeding period. The inset shows O_2_ consumption during feeding condition adjusted with body weight at 38.54 g using ANCOVA (HFD, AL/IF: *n* = 6/12). **(G)** Significant changes in O_2_ consumption between fasting and feeding periods in HFD-IF mice. Values are mean ± SEM; two-tailed unpaired and paired Student's *t*-test; ^*^P < 0.05 vs HFD-AL.

It has been suggested that IF promotes various health benefits, yet the underlying mechanism is still not fully understood. Given that adipose tissues are pivotal for supplying energy to other tissues during fasting, and were one of the most affected organs/tissues after IF, we performed transcriptome analysis by RNA sequencing (RNA-seq) on PWAT from mice subjected to IF or AL to gain insight into the genome-wide alteration of adipose tissue gene expression by IF. Excluding HFD-AL, the ND-AL, ND-IF and HFD-IF groups clustered together, indicating that even with HFD feeding, IF induced a global change in transcriptome that resembles gene expression profiles of metabolically normal animals (Figure 2A). Overall, 3 644 genes were differentially expressed among the four conditions and formed 6 distinct gene clusters. Clusters 1, 3, and 4 demonstrated significant gene enrichment by using terms of gene ontology (GO term) and KEGG pathway databases (Figure 2A; Supplementary information, Figure S4 and Table S1). In particular, we focused on clusters 1 and 3 that show dramatic differences in gene expression profile between HFD-AL and HFD-IF with clearly distinguished metabolic phenotypes. Cluster 1 genes (i.e., upregulated specifically in HFD-AL compared to other groups) were strongly enriched in the pathways involved in the immune response and inflammation, which are associated with insulin resistance and metabolic dysfunction^25^ (Figure 2A and Supplementary information, Figure S4A). qPCR verified that genes associated with inflammatory cells and their chemotactic activity were significantly downregulated in HFD-IF compared to HFD-AL (Figure 2B). On the other hand, GO term analysis of cluster 3 genes (i.e., downregulated specifically in HFD-AL) revealed biological processes including brown adipocyte differentiation (i.e., *Ebf2*)^26^ (Figure 2A and Supplementary information, Figure S4B). This finding is aligned with cluster 6 (i.e., upregulated only in HFD-IF group) indicating that the futile metabolic cycle with concurrently elevated genes involved in fatty acid synthesis (e.g., *Acaca*, *Scd1* and *Scd2*) and oxidation (e.g., *Cpt2*), as seen in β3-adrenergic receptor (β3-AR) agonist CL-316243- and cold-induced beige adipocytes^27,28^. Together, these results suggest that IF promotes a reduction of inflammation and an increase of thermogenic activities in WAT.

### IF leads to adipose thermogenesis

Next, we validated browning of WAT in HFD-IF mice. The expression of *Adrb3* (encoding β3-AR; indicative of sympathetic activation) as well as levels of beige/brown adipose markers (i.e., *Ppargc1a*, *Cidea* and *Ucp1*) were significantly elevated in PWAT of HFD-IF mice, compared to that of HFD-AL mice (Figure 2C). Similar results were also observed in WAT of post HFD-IF mice (Supplementary information, Figure S5A). In addition, *Ucp1* gene expression in BAT was found to be higher in HFD-IF mice compared to HFD-AL mice (Supplementary information, Figure S5B), suggesting increased BAT activation by IF. To visually examine beige adipocytes, we also utilized the beige-chaser mice generated by crossing *Ucp1-Cre* with *Rosa26^mT/mG^* mice. HFD-IF WAT exhibited more GFP^+^ beige adipocytes (Figure 2D), whereas the number of GFP^+^ brown adipocytes in BAT was indistinguishable between HFD-IF and HFD-AL mice (Supplementary information, Figure S5C). Consistent with the elevated level of beige adipocytes and higher *Ucp1* expression, indirect calorimetry, with normalization of body mass or adjustment for body mass using regression-based analysis of covariance (ANCOVA), revealed that O_2_ consumption in HFD-IF mice was increased, particularly during the feeding period (i.e., Day 2), without significant changes in physical activities, compared to HFD-AL mice (Figure 2E-2G; Supplementary information, Figure S5D-S5G). On the other hand, O_2_ consumption during the fasting period (i.e., Day 1) was not different between groups (Figure 2E and Supplementary information, Figure S5E). Together, these data suggest that IF led to an increase in beige adipocytes, thereby elevating energy expenditure, particularly during energy intake.

### Adipose thermogenesis largely contributes to IF-mediated metabolic benefits

To examine whether the beneficial effects of IF could be attributed to adipose thermogenesis, we performed IF in a thermoneutral (TN) condition (30 °C), where the thermogenic activation is significantly restricted^29^. Indeed, despite increased sympathetic activation (i.e., higher *Adrb3* expression), TN-HFD-IF mice showed no significant changes of beige/brown adipose marker gene expression in PWAT, compared to TN-HFD-AL mice (Supplementary information, Figure S6A). This suggests that thermoneutrality effectively hindered IF-induced WAT browning. Although *Ucp1* expression in BAT was increased by IF under TN conditions, its basal expression level was much lower (∼10%) compared to that at normal temperature (Supplementary information, Figure S6B). Consistently, O_2_ consumption was not significantly different between the two groups under TN feeding condition (Supplementary information, Figure S6C), suggesting that the increased Ucp1 in BAT of TN-HFD-IF mice may not be functionally significant. Intriguingly, however, TN-HFD-IF mice still exhibited a mild improvement in glucose tolerance, insulin sensitivity, and HOMA-IR, with a reduction in body weight, fat mass, as well as hepatic steatosis, compared to TN-HFD-AL mice (Supplementary information, Figure S6D-S6L). Although the TN condition does not completely abolish the metabolic benefits of IF, it significantly reduced the degree of improvement by IF (e.g., GTT difference: 9% in TN vs 28% in normal temperature), further emphasizing the key contributing role of adipose thermogenesis in IF.

### Fasting induces adipose-VEGF expression

**Figure 3:**
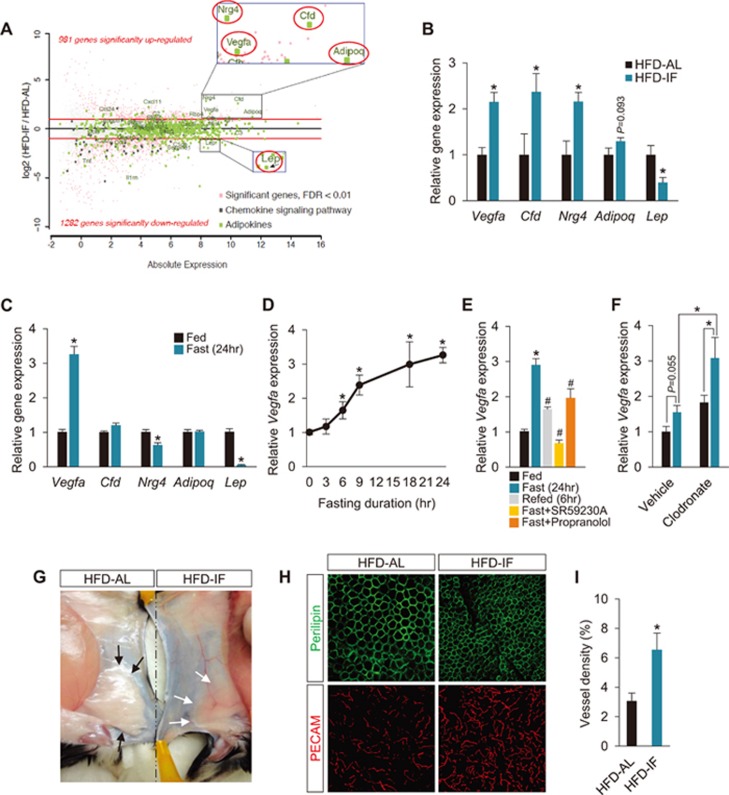
Fasting induces adipose-VEGF expression. **(A)** MA plot highlighting significantly altered mRNA expression of adipose-derived factors in PWAT of HFD-IF mice, compared to that of HFD-AL mice. *Vegfa*, *Cfd*, *Nrg4*, *Adipoq*, and *Lep* encode vascular endothelial growth factor, adipsin, neuregulin 4, adiponectin, and leptin, respectively. **(B)** qPCR validation of adipose-derived factors in PWAT (HFD, AL/IF: *n* = 6/8). **(C)** mRNA expression levels of *Vegfa*, *Cfd*, *Nrg4*, *Adipoq*, and *Lep* in PWAT at feeding and 24 h of fasting (*n* = 5 per group). **(D)** *Vegfa* mRNA expression in PWAT at different fasting durations (*n* = 5-6 per group). **(E)** *Vegfa* mRNA expression in PWAT at feeding, 24 h of fasting, 6 h of refeeding, fasting with β3-AR antagonist, SR59230A (5 mg/kg, i.p.), and fasting with non-specific β-AR antagonist, Propranolol (5 mg/kg, i.p.; *n* = 5 per group). **(F)** *Vegfa* mRNA expression in PWAT at feeding and 24 h of fasting with treatments of vehicle or clodronate (*n* = 5). **(G)** A representative macroscopic image illustrating increased vascularization in IWAT of HFD-IF mice, compared to HFD-AL mice. Black and white arrows indicate IWAT of HFD-AL and HFD-IF mice, respectively. **(H)** Representative microscopic images of adipocytes and blood vessels, visualized with perilipin and PECAM-1 antibodies, respectively, in whole-mount PWAT. **(I)** Quantification of vessel densities in PWAT. Data are mean ± SEM; one- or two-way ANOVA with Student-Newman-Keuls *post hoc* analysis and two-tailed unpaired Student's *t*-test; ^*^P < 0.05 vs HFD-AL or Fed. ^#^*P* < 0.05 vs Fast (24 h).

We next aimed to identify a molecular driver of IF-induced metabolic benefits and adipose thermogenesis. As WAT plays a crucial role in whole-body metabolic homeostasis through the production of numerous secretory proteins including adipokines^30,31^, we examined changes of the chemokine and adipokine pathways between HFD-AL and HFD-IF. Out of 710 adipose secretory proteins as defined by Lehr *et al.*^30^, several potential adipokine genes were markedly upregulated in HFD-IF PWAT, including *Vegfa* (VEGF), *Cfd* (adipsin), *Nrg4* (neuregulin 4), and *Adipoq* (adiponectin), whereas *Lep* (leptin), which positively correlates with adiposity, was downregulated (Figure 3A and 3B). To test whether these genes potentially function as drivers or are simply outcomes of repeated fasting, we examined how one-time fasting affects the expression of these genes. Noticeably, a 24-h fasting significantly increased *Vegfa* expression in WAT, while reducing *Lep* and *Nrg4* transcripts (Figure 3C). Given that higher expression of VEGF, leptin and neuregulin 4 positively correlates with adipose thermogenesis^8,9,32,33^, these results suggest that VEGF in WAT, but not leptin or Nrg4 (both decreased by fasting), is a potential modulator of IF.

We further examined how and where fasting-induced VEGF expression is regulated. *Vegfa* expression levels in WAT increase progressively with the fasting duration and the increase can be immediately reversed by refeeding (Figure 3D and 3E). Importantly, fasting-stimulated VEGF expression is restricted to WAT, not in BAT, other metabolic tissues or plasma (Supplementary information, Figure S7A-S7D). While previous studies showed that sympathetic activity controls VEGF expression^34^, total plasma catecholamine level was not increased by fasting (Supplementary information, Figure S7E), suggesting that fasting-stimulated VEGF expression in WAT is regulated via peripheral, not central, sympathetic nervous system. Moreover, fasting-induced VEGF was blocked markedly by the β3-AR antagonist, SR59230A, but less effectively by the non-specific β-AR antagonist, propranolol (Figure 3E). Given that β3-AR is known to be expressed specifically in adipocytes^35,36^, this result suggests that adipocytes may be the source of VEGF in response to sympathetic activation by fasting. To test this possibility, macrophages, which are another source of VEGF^37,38^, were depleted in mice by administration of Clodronate^37,39^. Notably, macrophage depletion, verified by abolished *F4/80* gene expression (Supplementary information, Figure S7F), did not affect fasting-mediated VEGF induction in WAT and was even increased in expression levels compared to vehicle-treated control mice (Figure 3F). This finding further supports our conclusion that adipocytes are the major sources of fasting-mediated VEGF induction. Functionally, the acute increase of adipose-VEGF by a 24-h fasting was not sufficient to induce changes in vascularization (Supplementary information, Figure S7G), whereas repeated fasting (i.e., IF) significantly increased WAT vascularization, both macroscopically and microscopically (Figure 3G-3I). Given that improved vascularization with adipose-derived VEGF plays a positive role in adipose tissue function and metabolic homeostasis against obesity and diabetes^6,7,8^, our results together suggest that fasting-induced adipose VEGF expression underlies IF-induced metabolic benefits and adipose thermogenesis.

### Adipose-VEGF is required for IF-mediated metabolic benefits

**Figure 4:**
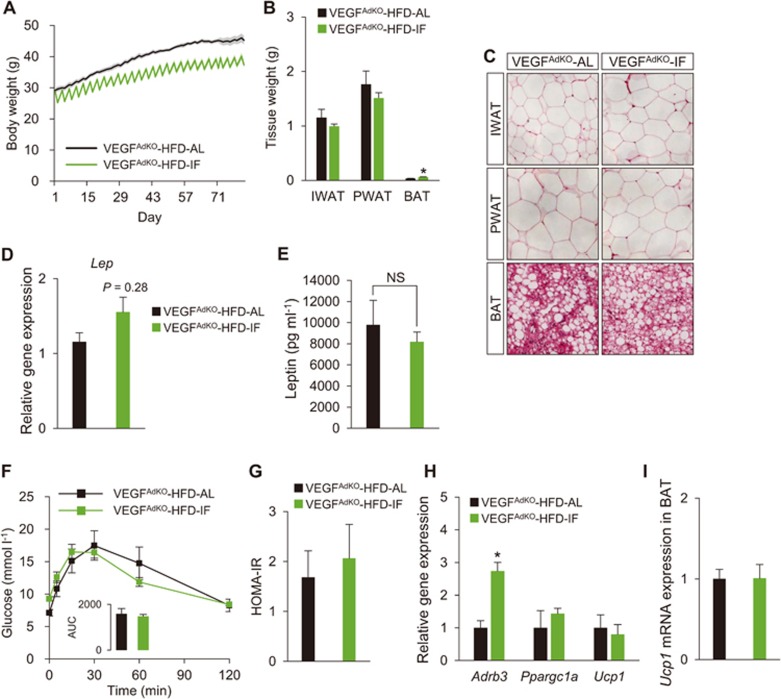
Adipose-VEGF is required for IF-mediated metabolic benefits. **(A)** Body weight measurements of *aP2-Cre*;*Vegfa^flox/flox^* mice (VEGF^AdKO^) subjected to AL and IF under HFD feeding (VEGF^AdKO^-HFD, AL/IF: *n* = 5/6). **(B)** Tissue weight of IWAT, PWAT, and BAT in VEGF^AdKO^-HFD-AL and -IF mice. **(C)** H&E-stained sections of IWAT, PWAT, and BAT show no noticeable differences between VEGF^AdKO^-HFD-AL and -IF mice. **(D)** *Lep* mRNA expression of PWAT in VEGF^AdKO^-HFD mice. **(E)** Plasma leptin levels. **(F)** GTT in VEGF^AdKO^-HFD mice. **(G)** HOMA-IR in VEGF^AdKO^-HFD mice. **(H)** Gene expression analysis revealed that IF increased sympathetic activation (*Adrb3*), but did not affect brown/beige adipocyte maker expression (i.e., *Ucp1*) in PWAT of VEGF^AdKO^ mice. **(I)** No changes in *Ucp1* expression in BAT of VEGF^AdKO^ mice upon IF. Data are mean ± SEM; two-tailed unpaired Student's *t*-test; ^*^P < 0.05 vs VEGF^AdKO^-HFD-AL. Lep, leptin.

We then investigated the implication of fasting-induced adipose-VEGF in the metabolic benefits of IF. Since VEGF generated by adipocytes and resident macrophages in adipose tissues could potentially influence and compensate each other, we utilized *aP2-Cre* mice that express Cre recombinase in preadipocytes, adipocytes, and macrophages^40^ to generate pan-adipose *Vegfa* KO mice (*aP2-Cre*;*Vegfa^flox/flox^*; hereafter VEGF^AdKO^), and conducted IF with this loss-of-function mouse model. Intriguingly, IF had no significant effect on WAT weight and adipocyte size in VEGF^AdKO^ mice fed HFD even with decreased body weight (Figure 4A-4C). Consistent with this, adipose *Lep* gene expression and plasma leptin levels, which positively correlate with adiposity, were also indistinguishable between VEGF^AdKO^-HFD-IF and VEGF^AdKO^-HFD-AL mice (Figure 4D and 4E). These data suggest that IF reduced body weight at the expense of lean mass. In addition, GTT and HOMA-IR revealed that IF does not improve glucose homeostasis in VEGF^AdKO^ mice (Figure 4F and 4G). IF-mediated improvement of liver steatosis was also abolished in VEGF^AdKO^ mice (Supplementary information, Figure S8A-S8C). Importantly, loss of adipose-VEGF abolished IF-mediated WAT browning, and there was no difference in *Ucp1* gene expression even in the presence of sympathetic activation (i.e., increased *Adrb3*; Figure 4H). Moreover, IF-induced BAT activation was not observed in VEGF^AdKO^ mice (Figure 4I). Together, these findings suggest that adipose-VEGF is required for IF-induced metabolic improvement and adipose thermogenesis.

### Intermittent adipose-VEGF overexpression is sufficient to mimic the IF-mediated metabolic benefits

**Figure 5:**
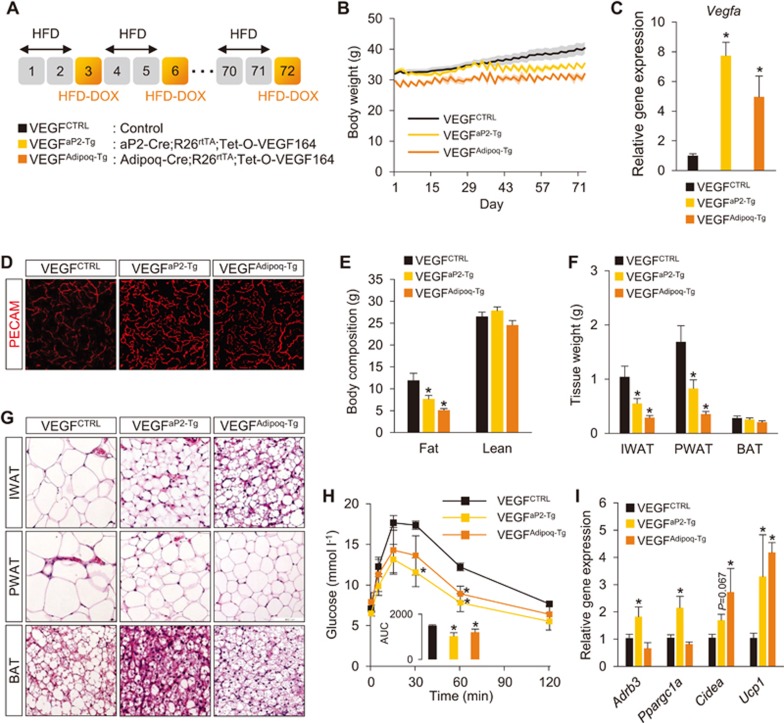
Intermittent adipose-VEGF overexpression is sufficient to mimic the IF-mediated metabolic benefits. **(A)** Schematic illustration of the 2:1 intermittent VEGF induction regimen and two different VEGF overexpression mouse models. **(B)** Body weight measurements during 10 weeks of adipose VEGF cyclic inductions. **(C)** *Vegfa* mRNA expression in PWAT at different fasting durations (*n* = 5-6 per group). **(D)** Representative microscopic images of PECAM-stained blood vessels in whole-mount PWAT. **(E)** Body composition showing fat and lean mass. **(F)** Tissue weight of IWAT, PWAT, and BAT. **(G)** H&E-stained sections of IWAT, PWAT, and BAT. **(H)** GTT. An insert graph shows AUC. **(I)** Gene expression analysis on WAT browning markers in PWAT. Data are expressed as mean ± SEM (VEGF^CTRL^: *n* = 10; VEGF^aP2-Tg^: *n* = 6; and VEGF^Adipoq-Tg^: *n* = 5); one- or two-way ANOVA with Student-Newman-Keuls *post hoc* analysis; ^*^P < 0.05 vs VEGF^CTRL^.

The results above prompted us to test whether periodic cycles of adipose-VEGF upregulation without fasting are sufficient to mimic the metabolic impact by IF. To mimic the fasting-mediated VEGF upregulation, two different lines of inducible adipose-VEGF mice (adipocytes + macrophage: *aP2-Cre*;*Rosa26^rtTA^*;*Tet-O-VEGF*, hereafter VEGF^aP2-Tg^; adipocytes: *Adipoq-Cre*;*Rosa26^rtTA^*;*Tet-O-VEGF*, hereafter VEGF^Adipoq-Tg^) were intermittently subjected to 45% HFD containing doxycycline (HFD-DOX), similar to IF cycle (Figure 5A). Importantly, both VEGF^aP2-Tg^ and VEGF^Adipoq-Tg^ mice under HFD-DOX cycles showed very similar metabolic phenotypes seen in HFD-IF mice, compared to control mice (VEGF^CTRL^; Figure 5B). Both transgenic mice showed significantly elevated adipose *Vegfa* expression and vascularization (Figure 5C and 5D; Supplementary information, Figure S9A). They also exhibited lower body weight due to decreased fat mass without changes of lean mass, reduced adipose tissue weight and cell size, and improved glucose homeostasis (Figure 5E-5H). Moreover, both transgenic mouse lines showed browning of WAT with elevated *Ucp1* and *Cidea* expression (Figure 5I), comparable to previous reports^6,7,8^, without BAT activation (Supplementary information, Figure S9B). Together with the data of adipose-specific *Vegfa* KO mice, these results illustrate the necessity and sufficiency of adipose-VEGF in IF-induced WAT browning and improved metabolism.

### Fasting and adipose-VEGF induce alternative activation of adipose macrophages

**Figure 6:**
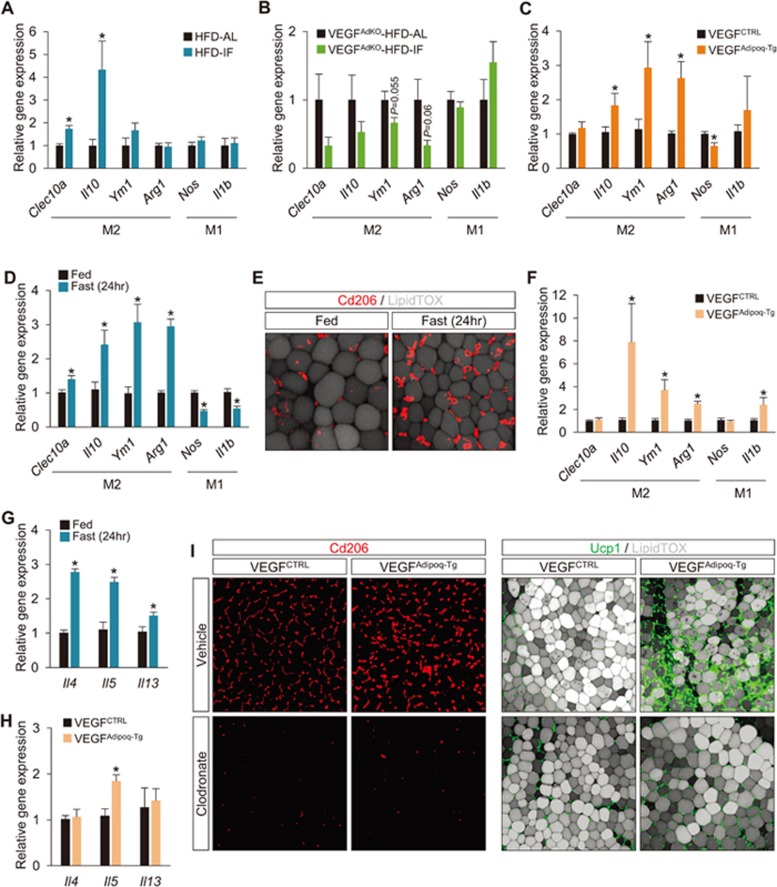
Fasting and adipose-VEGF induce alternative activation of macrophage. **(A)** M1/M2 macrophage marker gene expression analysis in HFD-AL and -IF mice. **(B)** M1/M2 macrophage marker gene expression in VEGF^AdKO^-HFD-AL and -IF mice. **(C)** M1/M2 macrophage marker gene expression after intermittent adipose-VEGF upregulation in VEGF^Adipoq-Tg^ mice. **(D)** M1/M2 macrophage marker gene expression analysis in fed and fasted (24 h) mice. **(E)** Representative images of M2 marker Cd206-stained cells in whole-mount PWAT of fed and fasted (24 h) mice. **(F)** M1/M2 macrophage marker gene expression analysis after acute adipose-VEGF upregulation in VEGF^Adipoq-Tg^ mice. **(G)** Type 2 cytokine gene expression in fed and fasted (24 h) mice. **(H)** Type 2 cytokine gene expression after acute adipose-VEGF upregulation (48 h) in VEGF^Adipoq-Tg^ mice. **(I)** Representative images of M2 macrophages and Ucp1 expression in WAT after acute adipose-VEGF upregulation in VEGF^Adipoq-Tg^ mice with treatments of vehicle or clodronate. Values are mean ± SEM (post HFD-AL: *n* = 7 and post HFD-IF: *n* = 9); two-tailed unpaired Student's *t*-test; ^*^P < 0.05 vs HFD-AL, VEGF^AdKO^-HFD-AL, VEGF^CTRL^ or fed mice. See also Supplementary information, Figure S7.

Although VEGF is implicated in WAT browning in response to cold, exercise, and environmental enrichment^9,10^, its underlying mechanism is not well understood. Recent studies have demonstrated that cold- and exercise-induced WAT browning is mediated through alternative activation of adipose macrophage by type 2 immune cytokines^41,42^. In addition, both cold acclimation and exercise, similar to our fasting condition, also increase adipose-VEGF expression^10,43^. Therefore, we speculated that browning of WAT in IF might be mediated by alternatively activated macrophage. Indeed, we found that IF cycles led to alternative activation of adipose tissue macrophage indicated by elevated expression of M2 macrophage marker genes (e.g., *Clec10a* and *Il10*) in PWAT (Figure 6A), whereas expression of classical M1 macrophage marker genes was unchanged by IF. As a result, the M1/M2 gene expression ratio was reduced by IF (Supplementary information, Figure S10A). On the contrary, but consistent with the lack of WAT browning in VEGF^AdKO^-HFD-IF mice, loss of adipose-VEGF completely eliminated the IF-mediated increase of alternatively activated adipose macrophage (Figure 6B; Supplementary information, Figure S10B and S10C). Similarly, the TN condition that blocked IF-stimulated WAT browning also hindered IF-induced M2 macrophage polarization (Supplementary information, Figure S10D and S10E). Notably, intermittent overexpression of adipose-VEGF using VEGF^Adipoq-Tg^ resulted in increased M2 marker gene expression, leading to a significant reduction in the M1/M2 ratio (Figure 6C; Supplementary information, Figure S10F and S10G). This finding was further verified by flow cytometry and whole-mount imaging, which showed that VEGF^Adipoq-Tg^ mice exhibited increased expression of Cd206 and Cd301 in WAT macrophage with a reduced M1/M2 ratio (i.e., Cd11c^+^/Cd206^+^ and Cd11c^+^/Cd301^+^), compared to VEGF^CTRL^ mice (Supplementary information, Figure S10H-S10J). Collectively, these data demonstrate that IF and adipose-VEGF cycles promote alternative activation of adipose macrophage closely linked to WAT browning.

Importantly, even one-time 24-h fasting is sufficient to induce M2 macrophage polarization as evidenced by increased M2 macrophages and their marker gene expression (e.g., *Clea10a*, *Il10*, *Ym1* and *Arg1*) as well as decreased expression of M1 marker genes (e.g., *Nos* and *Il1b*; Figure 6D and 6E; Supplementary information, Figure S11A). This suggests that these acute changes are repeated during IF, thereby facilitating its metabolic benefits. In addition, acute induction (i.e., 48 h) of adipose *Vegfa* overexpression (∼3.5-fold) using VEGF^Adipoq-Tg^ mice, whose level is comparable to 24-h fasted mice (Figure 3C), is sufficient to increase M2 macrophage (Figure 6F; Supplementary information, Figure S11B-S11E). However, direct administration of both mouse and human VEGF to Raw264.7 macrophages did not affect their properties, in contrast to interleukin-4 (IL-4) and lipopolysaccharide treatments that promote M2 and M1 polarizations, respectively (Supplementary information, Figure S11F). We found that a 24-h fasting upregulates gene expression of *Il-4*, *Il-5* and *Il-13* (Figure 6G), type 2 cytokines important for the activation of M2 macrophage^5^. Acute induction of adipose VEGF also elevated IL-5 gene expression (Figure 6H). These results suggest that VEGF-mediated M2 macrophage polarization is indirectly regulated, possibly via type 2 immune signaling.

Next, we tested whether adipose macrophages and their M2 polarization are required for VEGF-induced WAT browning. Consistent with a recent report^44^, acute induction of adipose VEGF in both VEGF^Adipoq-Tg^ and VEGF^aP2-Tg^ mice resulted in rapid browning of WAT with elevated Ucp1 expression (Figure 6I and Supplementary information, Figure S11G). However, this effect was markedly blocked by clodronate-induced macrophage depletion. Taken together, our results suggest that fasting-induced adipose-VEGF is a driver of M2 macrophage activation, which underlies IF-mediated adipose thermogenesis and associated metabolic benefits.

### VEGF expression in human WAT correlates with M2 macrophage and WAT browning

**Figure 7:**
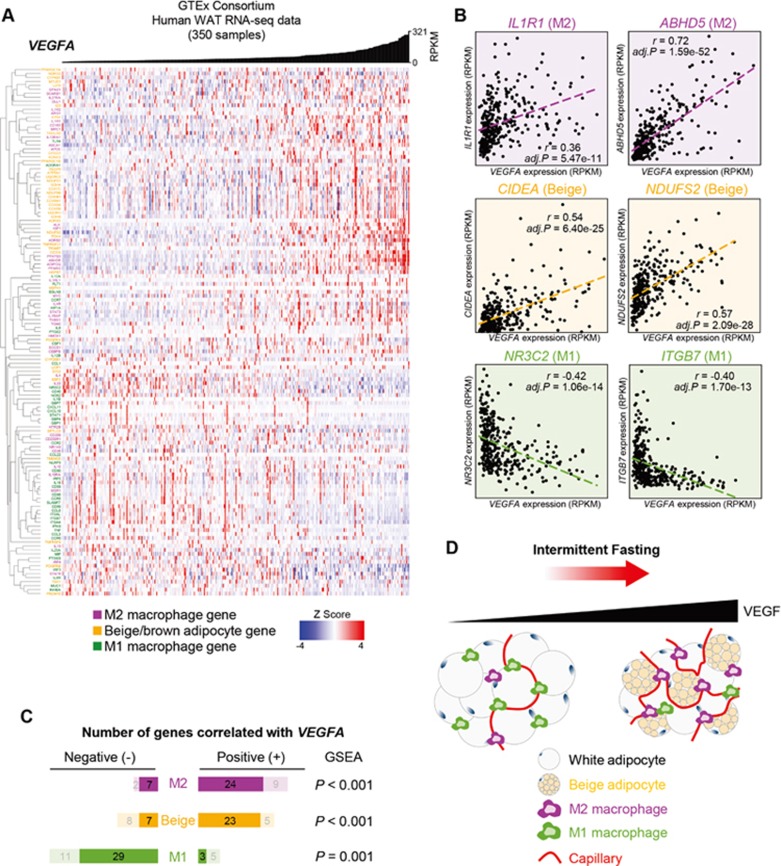
VEGF expression in human WAT correlates with M2 macrophage and WAT browning. **(A)** A correlation heatmap of *VEGFA* gene with unsupervised hierarchical clustering of M1/M2 macrophages- and beige/brown adipocyte-associated genes in human WAT. A histogram of *VEGFA* gene expression level (RPKM) is shown on the top of the heatmap. **(B)** Representative scatter plots showing correlation of *VEGFA* with *IL1R1* & *ABHD5* (M2), *CIDEA* & *NUDFS2* (beige), and *NR3C2* & *ITGB7* (M1) genes. **(C)** Summary of *VEGFA* correlation with M2, beige, and M1-associated genes. Permutation *P*-values with the GSEA are shown. **(D)** Schematic model of IF-mediated VEGF expression underlying adipose thermogenesis and M2 macrophage polarization. GSEA, gene set enrichment analysis.

To examine whether the adipose VEGF-M2 macrophage-WAT browning axis described above is relevant in humans, we utilized RNA-seq data of 350 human adipose tissues from the Genotype-Tissue Expression (GTEx) project^45^ to calculate the correlation between *VEGFA* gene expression and expression levels of M2-associated genes, M1-associated genes, and beige adipocyte-associated genes^46,47,48^ (Supplementary information, Table S2). The correlation heatmap using unbiased hierarchical clustering revealed that M2-associated genes positively correlated with *VEGFA* expression and were also clustered with beige adipocyte genes (Figure 7A). In contrast, most M1-associated genes negatively correlated with *VEGFA* expression. For example, as shown in the scatter plot, expression of the M2 macrophage genes *IL1R1* and *ABHD5*^49^, as well as the beige/brown adipocyte markers *CIDEA* and *NDUFS2*^50^, were positively correlated with *VEGFA* expression, whereas negative correlation with *VEGFA* was shown with the M1 macrophage genes *NR3C2* and *ITGB7*^51^ (Figure 7B). After applying the threshold of adjusted *P* < 0.05 in *VEGFA* correlation with the gene set enrichment analysis (GSEA), we identified 24 out of 31 M2-associated genes displaying a positive correlation with *VEGFA*, 23 out of 30 beige adipocyte genes displaying a positive correlation, and 29 out of 32 M1-associated genes displaying a negative correlation (Figure 7C and Supplementary information, Figure S12). Together with our mouse data, these results suggest that the adipose-VEGF expression level, which is elevated by IF, is not only associated with vasculature, but is also a metabolic index indicating M2 macrophage activation and beige adipocyte development in both humans and mice (Figure 7D).

## Discussion

Accumulating evidence suggests that IF provides various biological benefits in animal models and humans^13,14^. For example, the 5:2 diet, which involves CR for 2 non-consecutive days a week and unconstrained eating the other 5 days, has become a popular IF regimen and has a potential to be considered for medical interventions^13^. However, it remains elusive whether the effects upon IF, often tested by alternate day fasting in rodent models^19^, are attributed to reduced calorie intake and/or modified eating pattern. The present study, using an isocaloric mouse model with a 2:1 IF regimen, demonstrates calorie-independent metabolic benefits of IF. Together with recent important findings on time-restricted feeding and a fasting-mimicking diet (FMD)^52,53^, our data support the significance of not only 'what/how much' but also 'when/how often' in energy intake to sustain energy homeostasis and metabolic health. Our 2:1 IF rodent model provides an excellent platform for examining the mechanisms of isocaloric IF to prevent against various obesity-associated metabolic dysfunction.

Both IF and CR impact on nearly all metabolic organs/tissues including hypothalamus, liver, skeletal muscle, and adipose tissues^13,14^. However, the key organ/tissue driving the IF-induced metabolic benefits is not clearly defined. In the present study, our results posit that adipose tissues are not only an energy reservoir in fasting, but also play a pivotal role in IF-mediated metabolic benefits, particularly by regulating VEGF production. As sufficient vascularization is essential for adipose tissue development and function including cold-, exercise-, or environment-induced adaptive thermogenesis^9,10,11,54,55^, the activation of the adipose-VEGF pathway has been suggested as a new therapeutic strategy for the treatment of obesity and diabetes^56^. We demonstrate in this study that fasting is a physiological means of increasing adipose *Vegfa* gene expression (Figure 3), in addition to exercise^11^ and cold exposure^10^. Accordingly, IF confers similar metabolic impacts as physical exercise and cold exposure (e.g., browning of WAT) with markedly increased adipose vasculature. Our adipose VEGF loss- and gain-of-function mouse models further illustrate that repetition of fasting-induced adipose-VEGF expression underlies IF-mediated metabolic benefits. Interestingly, elevated VEGF expression was mainly noted in IWAT (i.e., subcutaneous WAT) or BAT of mice subjected to cold exposure^10,57^, whereas fasting-mediated augmentation of VEGF expression was greater in PWAT (i.e., visceral WAT) than in IWAT or BAT. Consistently, the profound weight loss in PWAT seen in our study (Supplementary information, Figure S1) and other study^52^ indicates that visceral WAT is apparently more sensitive to energy restriction. Together, these observations suggest that, although systemic metabolic outcomes by elevated VEGF expression in different adipose depots with different physiological stimuli appear to be comparable (i.e., improved glucose handling with elevated energy expenditure), subcutaneous WAT, visceral WAT, or BAT have their distinctive physiological and adaptive metabolic properties. This further suggests that combinatory stimuli to activate both subcutaneous and visceral WAT, such as IF and exercise or IF and cold exposure, could generate synergistic metabolic benefits.

It is now well appreciated that adipose thermogenesis, particularly browning of WAT, improves whole-body metabolism in response to various external and internal stimuli^3^, and that adipose-VEGF is clearly implicated in this process^6,7,8,9^. An improved supply of oxygen and nutrient via increased angiogenesis in adipose tissues can account for the necessity of adipose-VEGF in the adaptive responses to physiological stimuli and stress, but may not fully explain the sufficiency of adipose-VEGF in the induction of adipose thermogenesis as seen in several different transgenic mouse models. Of note, it appears that VEGF receptors, such as VEGFR1 (Flt1) and VEGFR2 (Flk1), are not expressed in preadipocytes or mature adipocytes^6^, leading us to postulate that VEGF acts indirectly in adipose tissue remodeling. Indeed, a recent study has demonstrated that adipose-VEGF induces endothelial PDGF-CC in response to β3-adrenergic activation, thereby stimulating PDGFRα^+^-progenitor beige adipocytes in WAT^58^. However, we did not find substantial changes of *Pdgfc* expression in the WAT of fasted or VEGF^Adipoq-Tg^ mice (data not shown). Importantly, our data and a recent study^59^ demonstrated that lifestyle interventions, IF, and CR triggered alternative activation (M2) of adipose macrophage, and the type 2 innate immune response that plays a critical role in this adipose thermogenic remodeling^5^. These immunological changes were also observed in mice with periodic overexpression of adipose-VEGF (driven by *Adipoq-Cre* and *aP2-Cre*), similar to a previous study^8^, but not in the mice lacking IF-mediated WAT browning due to thermoneutrality (TN-HFD-IF) or loss of adipose-VEGF (VEGF^AdKO^). In addition, both a one-time 24-h fasting and acute adipose-VEGF overexpression without fasting induce alternative activation of adipose macrophage, whereas depletion of macrophage in the presence of adipose-VEGF overexpression abrogated the WAT browning. Together, our results demonstrate that (1) M2 macrophage polarization in IF mice is not simply a consequence of leanness, but a driving force of adipose thermogenesis and metabolic benefits, and (2) adipose-VEGF plays a role in the regulation of M2 macrophage activation as a rapid adaptive response to energy restriction, likely even prior to angiogenesis. Furthermore, (3) the M1/M2 macrophage balance, rather than the number of macrophage, may be critical for adipose thermogenesis, as all fasted mice had reductions in the number of adipose macrophage regardless of conditions.

Nevertheless, little is known about the molecular mechanism underlying fasting- or adipose-VEGF-mediated M2 polarization. Our study showed that M2 macrophage polarization was indirectly regulated by VEGF, similar to the previous study^8^. What could be the mechanism of this VEGF-mediated M2 transition? Among various upstream signaling pathways implicated in M2 polarization^60^, we found elevated type2 cytokines by both fasting (IL-4, IL-5 and IL-13) and acute adipose-VEGF induction (IL-5), suggesting that adipose-VEGF may act on other upstream immune cells, such as eosinophils, T-helper cell 2 (Th2), or type 2 innate lymphoid cells (ILC2), to promote M2 polarization^61^. Therefore, future studies delineating a precise role (e.g., activation or recruitment of immune cells) and its molecular mechanism (e.g., through VEGFR1, VEGFR2, and VEGFB^56^) of VEGF signaling in adipose immunometabolism should uncover the fundamental mechanism in WAT browning. Furthermore, other adipose factors that we observed to be affected by IF, such as leptin, neuregulin 4, adiponectin and adipsin, could play a role in fasting-mediated immunometabolic responses. This might be particularly expected for leptin, whose expression level is reduced by IF or fasting in WAT and BAT (not in skeletal muscle or liver). Leptin modulates a wide range of immune and inflammatory processes, and leptin-deficient mast cells undergo M2 polarization^62^. Given that leptin could synergistically act with VEGF (e.g., in angiogenesis)^63^, it will be of interest to examine the implication of leptin in fasting- and VEGF-mediated metabolic benefits. On the other hand, these adipose factors may be involved in the non-thermogenic effects of IF. Although thermoneutrality markedly reduced the metabolic benefits of IF with inhibition of WAT browning, it did not completely eliminate the IF's effects as adipose-derived factors showed similar gene expression (data not shown). As previous studies demonstrate that neuregulin 4 and adipsin are implicated in inhibition of hepatic steatosis^32^ and improvement of insulin secretion^64^, respectively, the non-thermogenic benefits of IF may be attributed to these adipose-derived factors or possibly other physiological responses, such as reduced adipose inflammation and increased insulin sensitivity (Figure 2A, Cluster #3).

IF has been practiced in human clinical settings with various fasting regimens, and most studies revealed similar beneficial health effects such as body weight reduction and increased insulin sensitivity^18,21,65,66^. However, IF can be stressful to body and mind, because 24-h fasting can reduce body weight by 2%-3% in humans^65^ and 5%-10% in mice. Although it is not easy to compare the effects of fasting in mice directly with humans due to differences in basal metabolic rate, food intake, as well as age differences^67^, IF appears to improve eating behavior and mood in humans^68^. It will be important to overcome potential limitations by developing a method to improve the practicality of IF if our findings are to have translational perspective. For example, a method like FMD that provides various metabolic benefits in both mice and humans^18,21,66^ would improve the practicality of IF in a clinical setting. Further rigorous studies are required to examine: (1) whether the beneficial effects of IF last after stopping the fasting (i.e., if there is a rebound effect); (2) whether there is any potential harm of IF; and (3) whether IF benefits apply to all generations (i.e., an age-dependent issue) and disease conditions. Notably, 5 weeks of IF was able to drive beneficial metabolic effects in 8-month-old mice with HFD-induced obesity (unpublished data), further emphasizing the therapeutic potential of IF in age-associated metabolic abnormalities. To further determine the mechanism of IF, it is necessary to test whether fasting (or IF) induces VEGF expression in human adipose tissues, and whether fasting-induced adipose VEGF promotes M2 polarization and WAT browning in humans. Thus, future studies are warranted to examine the adaptive response of human adipose tissues to fasting or IF intervention via thermogenic and even non-thermogenic pathways, in association with the proposed mechanisms such as circadian rhythm^53,69^, gut microbiome changes^70^, and sleep regulation.

In summary, our data demonstrate that metabolic benefits of IF are largely mediated by adipose thermogenesis without overall caloric reduction. We showed that fasting-induced adipose-VEGF plays a key role in WAT browning through M2 macrophage activation. This finding is further supported by human adipose tissue gene expression analysis illustrating positive correlations between adipose-VEGF expression and both M2 macrophage and WAT browning. Together, the present study illustrates the role of IF-induced adipose-VEGF in remodeling the immunometabolic property of adipose tissue, highlighting the importance of eating pattern and physiological fasting duration to sustain metabolic homeostasis.

## Materials and Methods

### Animals

All animal experimental protocols approved by the Animal Care Committee of the Centre of Phenogenomics conformed to the standards of the Canadian Council on Animal Care. No statistical methods were used to predetermine sample size. The investigators were not blinded to allocation during experiments. The colony was housed in a specific pathogen-free (SPF) facility in ventilated cages with controlled environment settings (21-22 °C, 30%-60% humidity for normal housing), 12-h light/dark cycles, and free access to water. For the thermoneutrality experiment, mice were housed at 30 °C. C57BL/6J mice were purchased from the Jackson Laboratory. *aP2-Cre*, *Adipoq-Cre*, *Ucp1-Cre*, *Vegfa^flox/flox^*; *TetO-Vegf164*, *Rosa26^rtTA^* and *Rosa26^mT/mG^* mice were described previously^6,71,72^. Age-matched littermate controls were used for each experiment.

### IF regimen and diets

Body weight-matched 8-week-old male C57BL/6J, VEGF^AdKO^, and *Ucp1-Cre*;*Rosa26^mT/mG^* mice were randomly divided into two groups: AL and IF groups. Mice were fed either ND (Harlan #2918; 17% fat) or 45% HFD (#D12451, Research Diets). Mice in the IF group were subjected to the 2:1 IF regimen, comprising 1 day of fasting followed by 2 days of feeding; the food was removed at 12:00 PM and then provided again the following day (24 h later) at 12:00 PM. Mice in the AL group were handled equivalently. To induce periodic VEGF overexpression in adipose tissues, VEGF^CTRL^, VEGF^aP2-Tg^, and VEGF^Adipoq-Tg^ mice were subjected to 45% HFD-DOX (Research Diets) once every 3 days (Figure 5A).

### Statistical analysis

All results are presented as mean ± SEM. Statistical significance of differences among groups was determined by two-tailed unpaired and paired Student's *t*-test as well as analysis of variance (ANOVA) with *post hoc* analysis, Student-Newman-Keuls, using Sigma Stat (SPSS) or PRISM 5.0 (GraphPad). Differences with *P* < 0.05 were considered statistically significant.

Other methods are described in Supplementary information, Data S1.

## Author Contributions

K-HK, C-CH, and H-KS conceived, designed, and supervised the research project. K-HK, YHK, JES, JHL, JHM, SK, MSC, K-OD and J-AY performed mouse metabolic experiments. K-HK and YHK analyzed mouse metabolic data. K-HK, YHK, and JHL performed gene expression analysis with assistance from SK and MSC. Histology was conducted by JZ, and analyzed by K-HK, YHK, KF, and FL. JES performed whole-mount adipose tissue staining for blood vessel and macrophage. JGP and SMIH analyzed mouse RNA-seq data. JGP and K-HK performed human gene correlation analysis. AN, AK, and J-RK provided scientific discussion and technical support. K-HK, C-CH, and H-KS wrote the manuscript. All authors discussed the results and commented on the manuscript.

## Competing Financial Interests

The authors declare no competing financial interests.

## Supplementary Information

Supplementary information, Figure S1 Feeding analyses after 24-h fasting and metabolic phenotypes of mice subjected to IF.

Supplementary information, Figure S2 The IF-mediated metabolic benefits are independent of energy intake – pair feeding study.

Supplementary information, Figure S3 IF provides therapeutic effects against preexisting obesity and its metabolic dysfunction.

Supplementary information, Figure S4 Pathway and GO term analyses on WAT upon IF.

Supplementary information, Figure S5 IF induces adipose thermogenic activity without affecting physiological activity.

Supplementary information, Figure S6 Metabolic phenotypes of mice subjected to IF under the thermoneutral condition.

Supplementary information, Figure S7 Fasting induces adipose-specific VEGF upregulation.

Supplementary information, Figure S8 Adipose-VEGF is required for IF-mediated metabolic benefits.

Supplementary information, Figure S9 Intermittent VEGF overexpression in BAT did not increase thermogenic activity in BAT.

Supplementary information, Figure S10 IF and adipose-VEGF overexpression induces alterative activation of macrophages.

Supplementary information, Figure S11 24-h fasting and acute adipose-VEGF induction lead to alterative activation of adipose macrophages.

Supplementary information, Figure S12 VEGF expression in human WAT correlates with M2 macrophage and WAT browning.
