# Supplementary material for: Intermittent fasting promotes adipose thermogenesis and metabolic homeostasis via VEGF-mediated alternative activation of macrophage
Source: Cell Res. 2017 Oct 17;27(11):1309–26. doi: 10.1038/cr.2017.126 (PMC5674160; doi:10.1038/cr.2017.126)
Supplement: Supplementary information, Figure S1 — Feeding analyses after 24-h fasting and metabolic phenotypes of mice subjected to IF. [file cr2017126x1.pdf]

Supplementary information, Figure S1

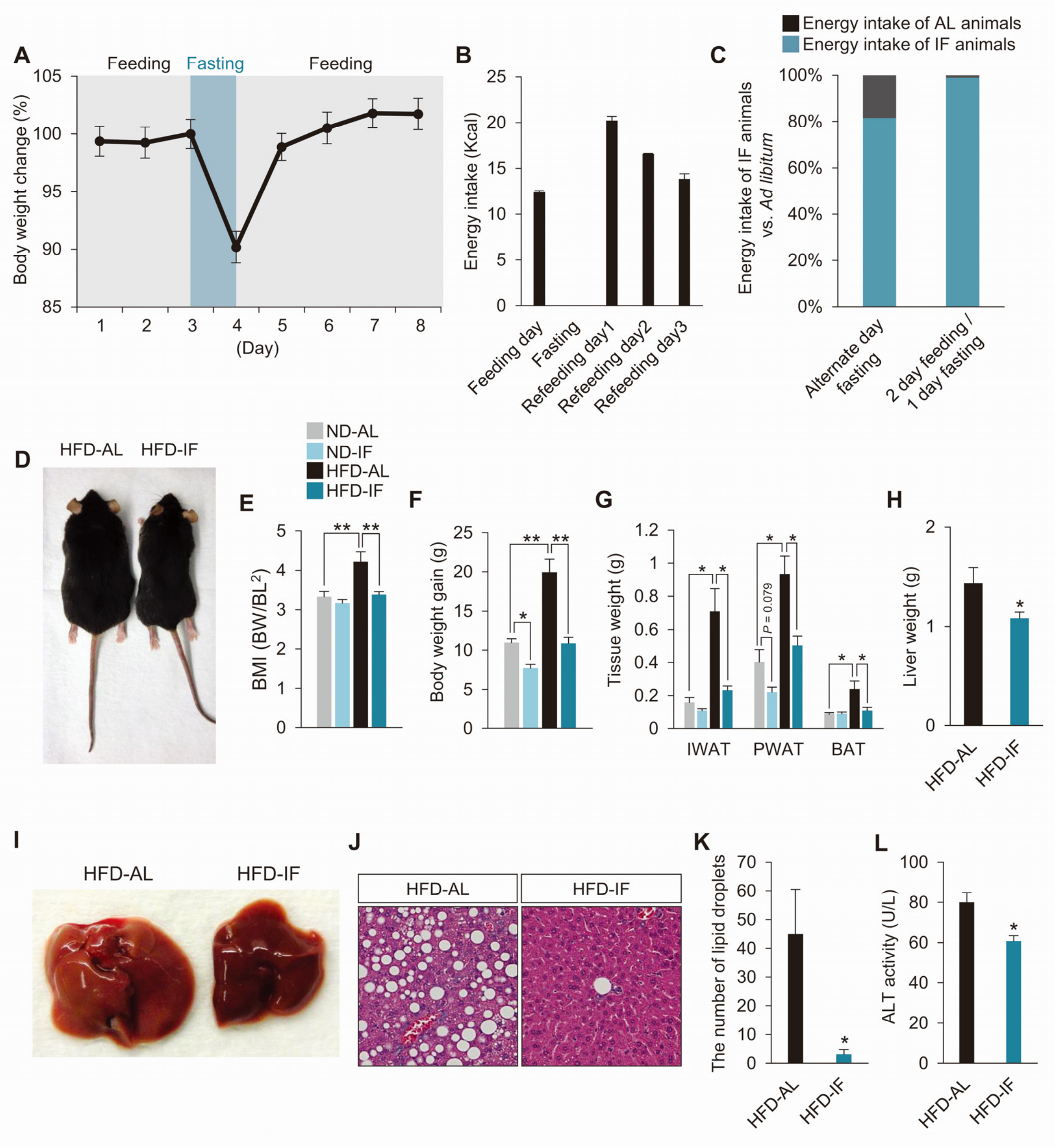

Figure S1 Feeding analyses after 24-h fasting and metabolic phenotypes of mice subjected to IF. (A) Daily body weight changes of mice before and after 24-h fasting (n = 10). **(B)** Daily energy intake before and after 24-h fasting (n = 5 cages; 2 mice per cage). **(C)** Comparison of energy intakes between alternate day fasting (i.e., 1 day feeding-1 day fasting, 1:1 IF) and 2:1 intermittent fasting (i.e., 2 day feeding-1 day

fasting). In 1:1 IF regime, only 85% of food intake was compensated during the 1 day of the refeeding period compared to the amount taken for 2 days of feeding condition. On the other hand, 99% of energy intake was achieved when 2 days of refeeding period was given, compared to 3 days of feeding condition. **(D)** A representative image of mice subjected to high-fat diet (HFD) *ad libitum* (AL) or HFD-IF. **(E)** Body mass index (ND-AL: n = 7; ND-IF: n = 8; HFD-AL: n = 7; and HFD-IF: n = 8). **(F)** Body weight gain after 16 weeks of IF cycles. **(G)** Tissue weights of inguinal and periogonadal WAT (IWAT and PWAT) as well as brown adipose tissue (BAT). **(H)** Liver weights. **(I)** Representative images of liver from HFD-AL and HFD-IF mice. **(J)** Histological sections of HFD-AL and HFD-IF mouse liver. **(K)** The number of lipid droplets in liver. **(L)** Plasma alanine aminotransferase (ALT) activity. Values are mean ± SEM; two-way ANOVA with Student-Newman-Keuls *post-hoc* analysis and two-tailed unpaired Student's *t*-test; *$P < 0.05$ and ** $P < 0.01$ vs. HFD-AL.
