# Supplementary material for: Intermittent fasting promotes adipose thermogenesis and metabolic homeostasis via VEGF-mediated alternative activation of macrophage
Source: Cell Res. 2017 Oct 17;27(11):1309–26. doi: 10.1038/cr.2017.126 (PMC5674160; doi:10.1038/cr.2017.126)
Supplement: Supplementary information, Figure S2 — The IF-mediated metabolic benefits are independent of energy intake – pair feeding study. [file cr2017126x2.pdf]

Supplementary information, Figure S2

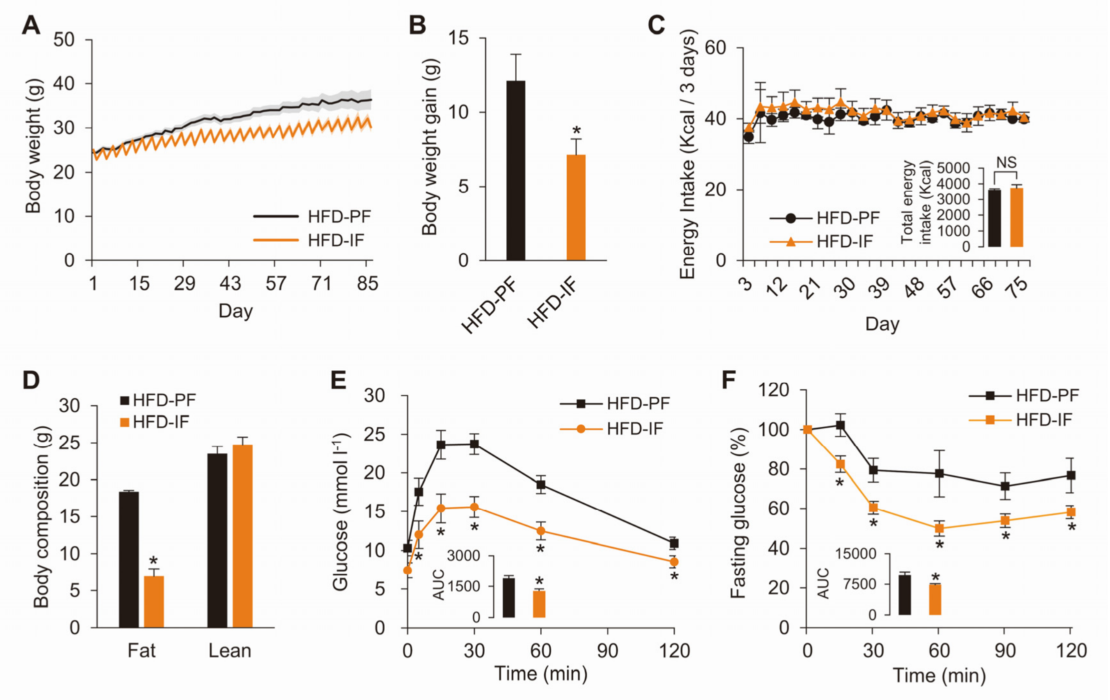

Figure S2 The IF-mediated metabolic benefits are independent of energy intake – pair feeding study. (A) Body weight measurement during 12 weeks of IF compared to PF mice fed HFD. **(B)** Body weight gain. **(C)** Changes of energy intake during 12 weeks of IF and pair-feeding (PF) cycles. An inset shows accumulated energy intake during the IF cycles. **(D)** Body composition showing fat and lean mass. **(E)** GTT in HFD-PF and HFD-IF mice. An inset graph shows AUC. **(F)** ITT in HFD-PF and HFD-IF mice. An inset graph shows AUC. (PF: n = 6 and IF: n = 6); two-tailed unpaired Student's *t*-test; *$P < 0.05$ vs. HFD-PF. NS, not significant.
