# Supplementary material for: Intermittent fasting promotes adipose thermogenesis and metabolic homeostasis via VEGF-mediated alternative activation of macrophage
Source: Cell Res. 2017 Oct 17;27(11):1309–26. doi: 10.1038/cr.2017.126 (PMC5674160; doi:10.1038/cr.2017.126)
Supplement: Supplementary information, Figure S3 — IF provides therapeutic effects against preexisting obesity and its metabolic dysfunction. [file cr2017126x3.pdf]

Supplementary information, Figure S3

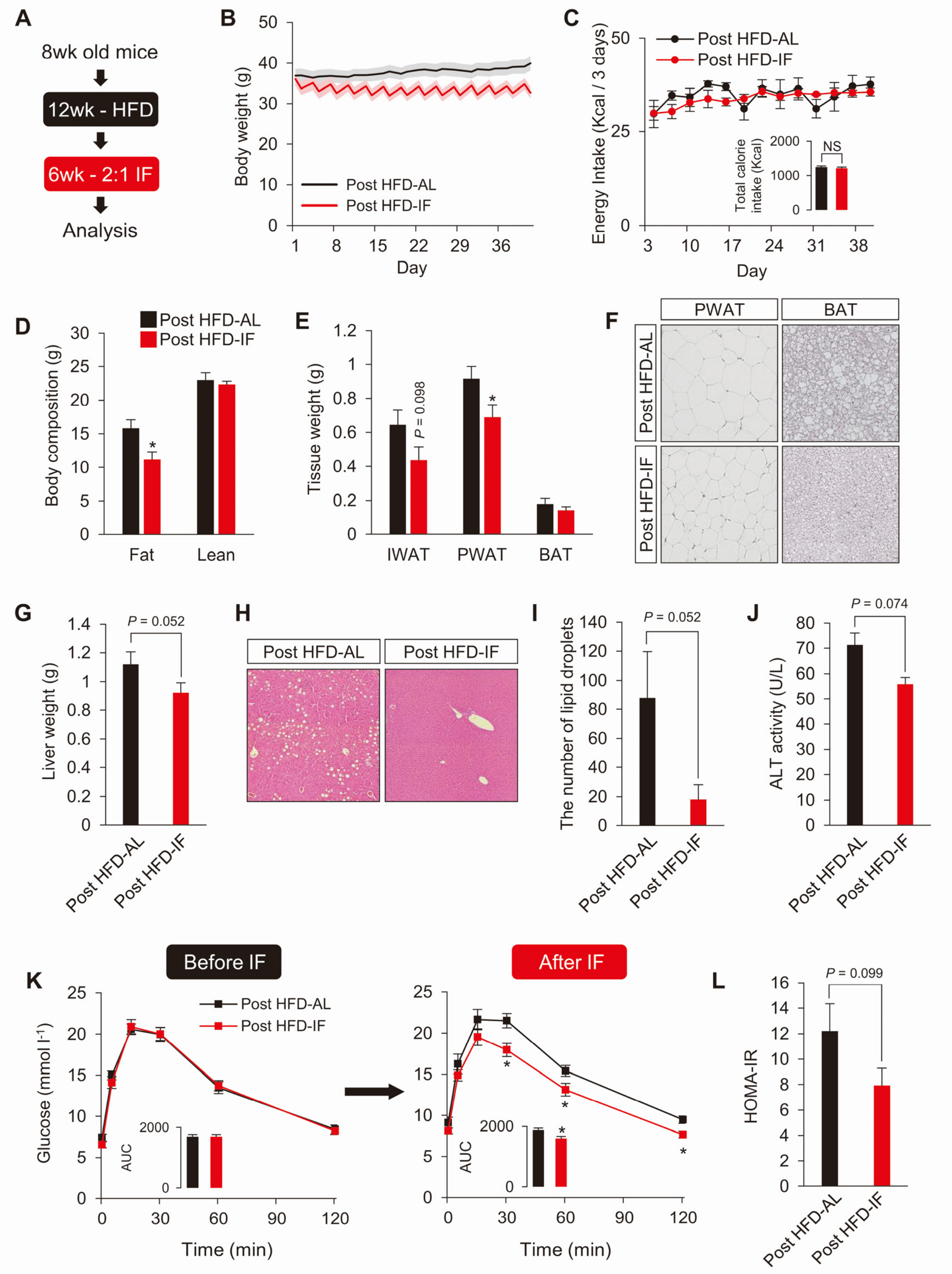

Figure S3 IF provides therapeutic effects against preexisting obesity and its

metabolic dysfunction. (A) Schematic illustration of Post-HFD-IF experiment. **(B)** Body weight measurements of diet-induced obese mice upon AL an IF treatments. **(C)** No difference in caloric intake between Post HFD-AL and -IF mice. An inset shows total caloric intake. **(D)** Body composition analysis showed specific reduction in fat mass without affecting lean mass. **(E)** Weights of adipose tissues; IWAT, PWAT, and BAT. **(F)** Histology of PWAT and BAT. **(G)** Liver weight. **(H)** Representative H&E stained sections of liver. **(I)** The number of lipid droplet in liver. **(J)** ALT activity. **(K)** Improved GTT after 6 weeks of IF. An inset graph shows area under curve (AUC). **(L)** Homeostatic model assessment of insulin resistance (HOMA-IR). Values are mean ± SEM (Post HFD-AL: n = 7 and Post HFD-IF: n = 9); two-tailed unpaired Student's *t*-test; *$P < 0.05$ vs. Post-HFD-AL. NS, not significant.
