# Supplementary material for: Intermittent fasting promotes adipose thermogenesis and metabolic homeostasis via VEGF-mediated alternative activation of macrophage
Source: Cell Res. 2017 Oct 17;27(11):1309–26. doi: 10.1038/cr.2017.126 (PMC5674160; doi:10.1038/cr.2017.126)
Supplement: Supplementary information, Figure S4 — Pathway and GO term analyses on WAT upon IF. [file cr2017126x4.pdf]

Supplementary information, Figure S4

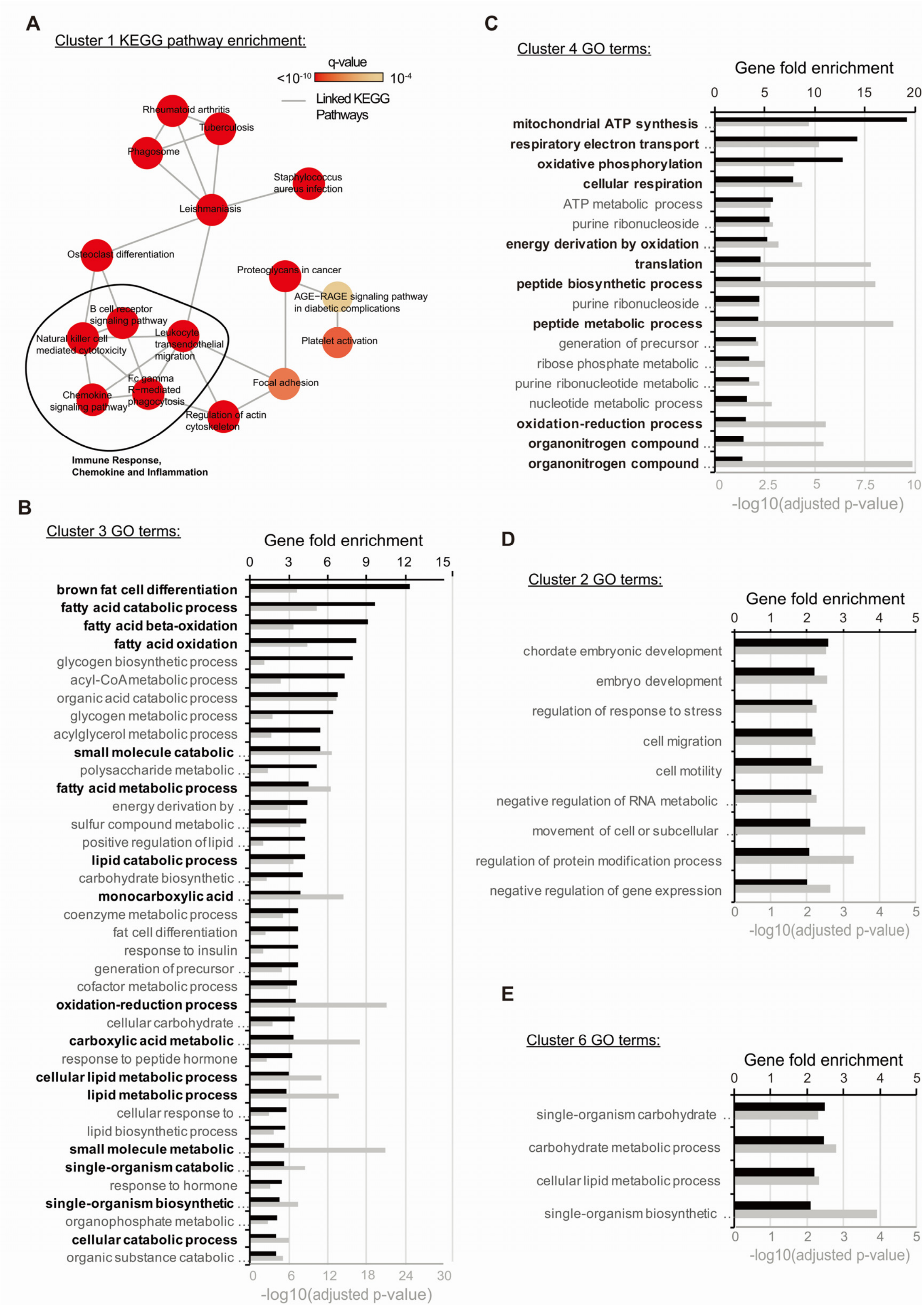

Figure S4 Pathway and GO term analyses on WAT upon IF. (A) KEGG pathway enrichment of the cluster 1 (i.e., higher in HFD-AL mice). **(B)** GO term analysis of the cluster 3 (i.e., lower in HFD-AL mice). **(C)** GO term analysis of the cluster 4 (i.e., higher in ND mice). **(D)** GO term analysis of the cluster 2 (i.e., lower in HFD-IF mice). **(E)** GO term analysis of the cluster 6 (i.e., higher in HFD-IF mice). GO terms with adjusted *P*-values $< 10^{-6}$ are in bold. Adjusted *P*-values were calculated based on Bonferroni correction method.
