# Supplementary material for: Intermittent fasting promotes adipose thermogenesis and metabolic homeostasis via VEGF-mediated alternative activation of macrophage
Source: Cell Res. 2017 Oct 17;27(11):1309–26. doi: 10.1038/cr.2017.126 (PMC5674160; doi:10.1038/cr.2017.126)
Supplement: Supplementary information, Figure S5 — IF induces adipose thermogenic activity without affecting physiological activity. [file cr2017126x5.pdf]

Supplementary information, Figure S5

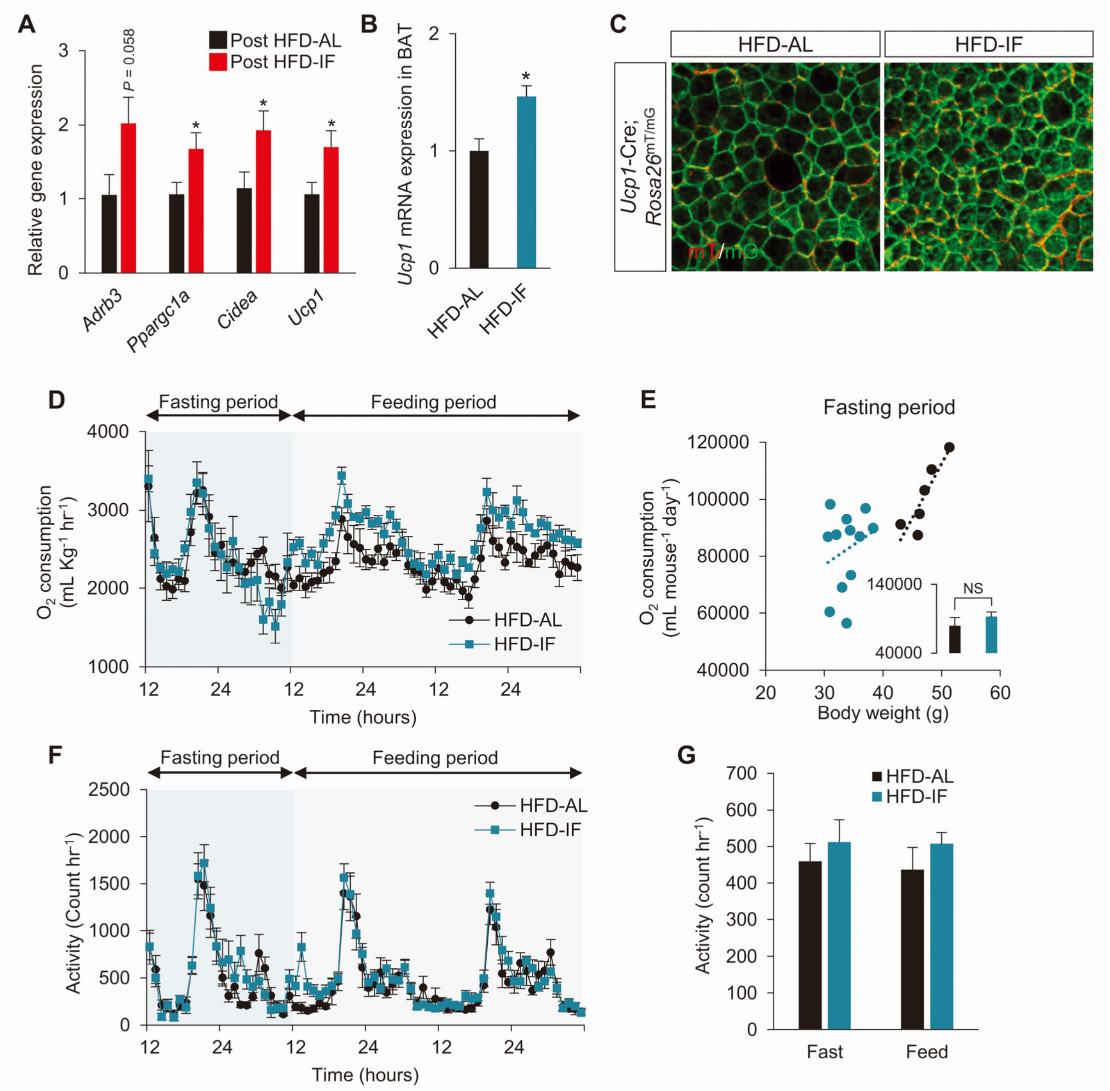

Figure S5 IF induces adipose thermogenic activity without affecting physiological activity. (A) Gene expression analysis on WAT browning markers in Post-HFD-IF mice compared to Post-HFD-AL mice. **(B)** Increased *Ucp1* expression in BAT of HFD-IF mice compared to HFD-AL mice. **(C)** Representative images of whole-mount BAT of brown/beige chaser (*Ucp1-Cre Rosa26*$^{mT/mG}$) mice subjected to AL and IF under HFD feeding. GFP^{+} cells indicate brown adipocytes in BAT. **(D)** Traces of O_2 consumption normalized with body weight during one IF cycle (i.e., 2 day feeding-1 day fasting). **(E)**

Linear regression analysis of O_2 consumption as a function of body weight during fasting period indicates no difference in energy expenditure between HFD-AL and HFD-IF. An inset graph shows O_2 consumption during fasting condition, adjusted with body weight at 38.54 g using ANCOVA (HFD, AL/IF: n = 6/12). N.S., not significant. **(F)** Traces of physical activities of HFD-AL and HFD-IF mice during one IF cycle. **(G)** Average of physical activities per hour during fasting and feeding periods. Data are mean ± SEM; two-tailed unpaired Student's *t*-test; *, $P < 0.05$ vs. Post HFD-AL or HFD-AL.
