# Supplementary material for: Intermittent fasting promotes adipose thermogenesis and metabolic homeostasis via VEGF-mediated alternative activation of macrophage
Source: Cell Res. 2017 Oct 17;27(11):1309–26. doi: 10.1038/cr.2017.126 (PMC5674160; doi:10.1038/cr.2017.126)
Supplement: Supplementary information, Figure S6 — Metabolic phenotypes of mice subjected to IF under the thermoneutral condition. [file cr2017126x6.pdf]

Supplementary information, Figure S6

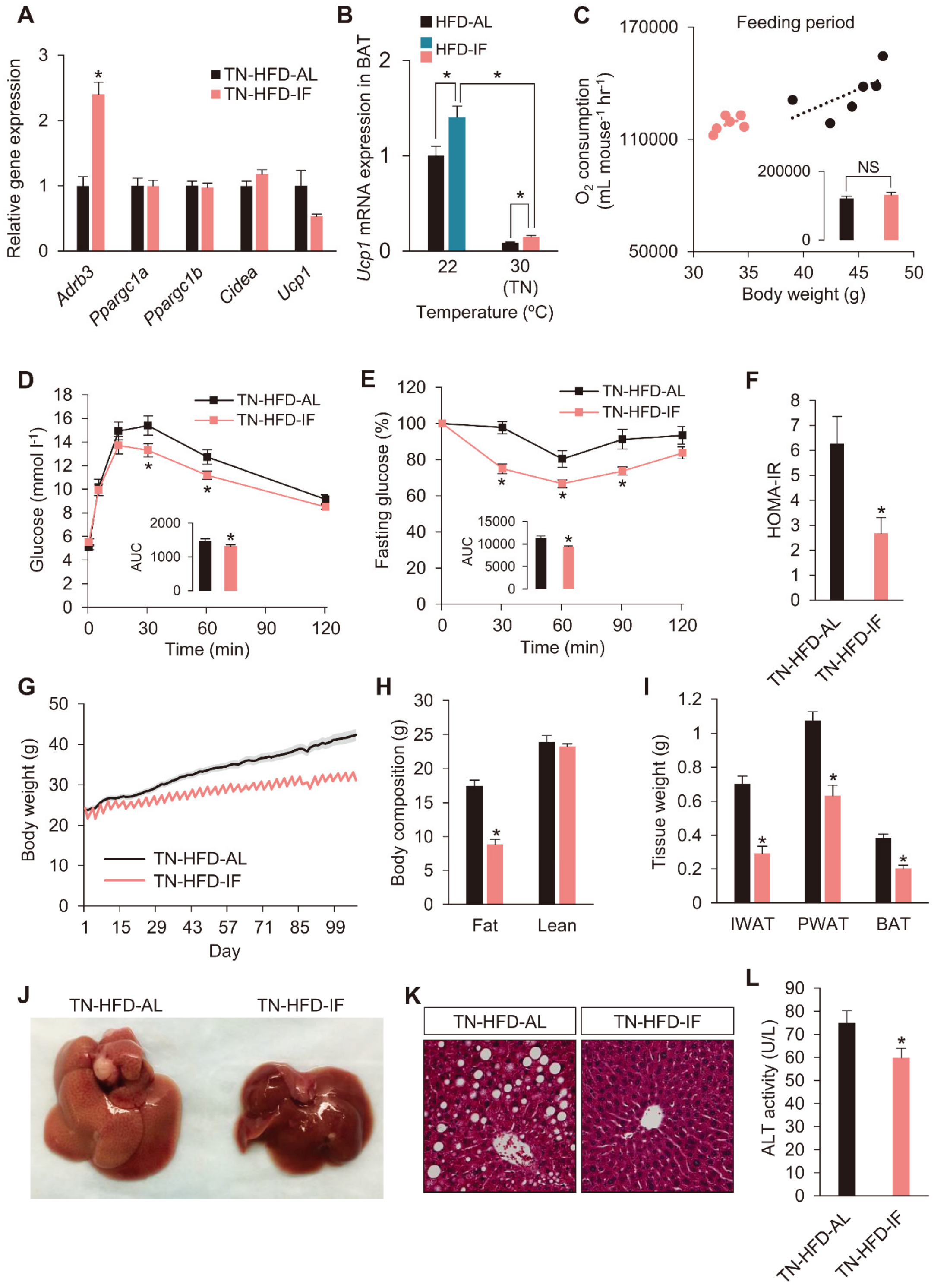

Figure S6 Metabolic phenotypes of mice subjected to IF under the thermoneutral condition. (A) Marker gene expression of sympathetic activation and brown/beige adipocyte in PWAT of HFD-IF mice at thermoneutrality (TN; 30 ºC) (TN-HFD, AL/IF:

n = 11/12). **(B)** *Ucp1* expression in BAT at TN, which was increased by IF compared to TN-HFD-AL mice, was significantly lower, compared to HFD-IF mice at normal temperature (22 ºC). **(C)** O_2 consumption during feeding condition as a function of body weight showed no differences between TN-HFD-AL and TN-HFD-IF mice (n = 6 per group). An inset graph shows O_2 consumption adjusted with body weight at 38.67 g using ANCOVA. **(D)** Although it was improved, GTT showed that IF at 30 ºC resulted in reduced protective effects, compared to HFD-IF at 22 ºC. An inset graph shows area under curve (AUC). **(E)** ITT. An inset graph shows AUC. **(F)** HOMA-IR. **(G)** Body weight measurements. **(H)** Body composition showing fat and lean mass. **(I)** Weights of adipose tissues; IWAT, PWAT, and BAT. **(J)** Representative image of liver from TN-HFD-AL and TN-HFD-IF mice. **(K)** Histological sections of liver. **(L)** Plasma alanine aminotransferase (ALT) activities. Values are mean ± SEM; two-tailed unpaired and paired Student's *t*-test; *$P < 0.05$ vs. TN-HFD-AL. N.S., not significant.
