# Supplementary material for: Intermittent fasting promotes adipose thermogenesis and metabolic homeostasis via VEGF-mediated alternative activation of macrophage
Source: Cell Res. 2017 Oct 17;27(11):1309–26. doi: 10.1038/cr.2017.126 (PMC5674160; doi:10.1038/cr.2017.126)
Supplement: Supplementary information, Figure S7 — Fasting induces adipose-specific VEGF upregulation. [file cr2017126x7.pdf]

Supplementary information, Figure S7

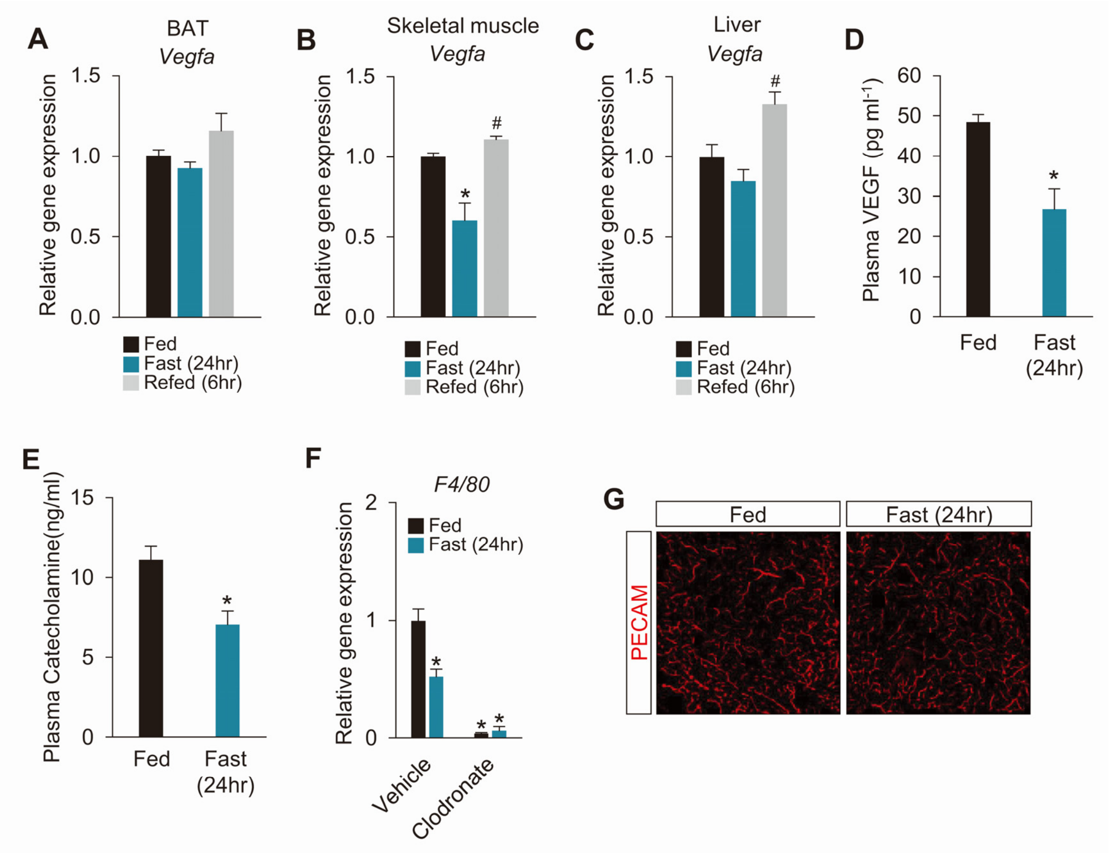

Figure S7 Fasting induces adipose-specific VEGF upregulation. (A-C) *Vegfa* mRNA expression in BAT (A), skeletal muscle (B; i.e., quadriceps femoris), and liver (C) at feeding, 24 h fasting, and 6 h refeeding (n = 5 per group). **(D)** Plasma VEGF levels at feeding and 24 h fasting. **(E)** Plasma catecholamine levels at feeding and 24 h fasting. **(F)** F4/80 gene expression verifying depletion of adipose macrophage upon clodronate injection. **(G)** Representative microscopic images of PECAM-stained blood vessels in whole-mount PWAT of fed and fasted (24 h) mice. Data are mean ± SEM; one-way ANOVA with Student-Newman-Keuls *post-hoc* analysis and two-tailed unpaired Student’s *t*-test; $*P < 0.05$ vs. Fed. $^{\#}P < 0.05$ vs. Fast (24 h).
