# Supplementary material for: Intermittent fasting promotes adipose thermogenesis and metabolic homeostasis via VEGF-mediated alternative activation of macrophage
Source: Cell Res. 2017 Oct 17;27(11):1309–26. doi: 10.1038/cr.2017.126 (PMC5674160; doi:10.1038/cr.2017.126)
Supplement: Supplementary information, Figure S8 — Adipose-VEGF is required for IF-mediated metabolic benefits. [file cr2017126x8.pdf]

Supplementary information, Figure S8

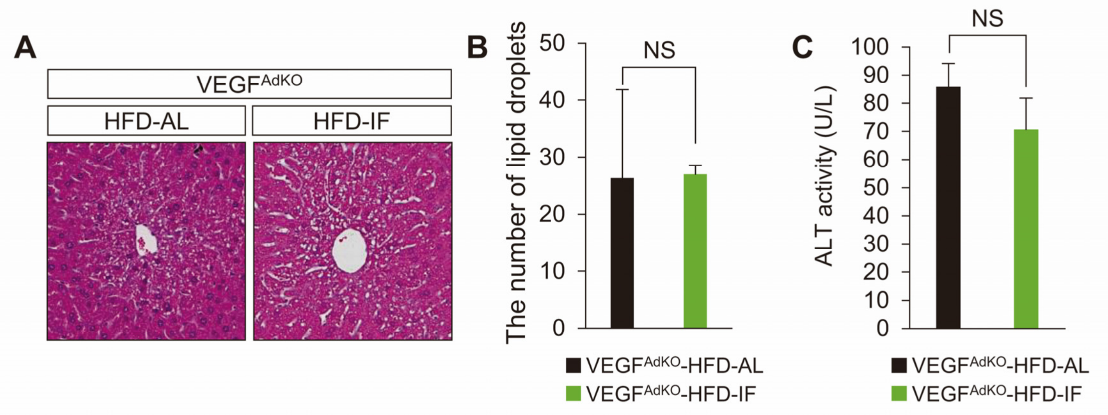

Figure S8 Adipose-VEGF is required for IF-mediated metabolic benefits. (A) Histological sections of VEGF^{AdKO}-HFD-AL and -IF mouse liver. **(B)** The number of lipid droplets in liver of VEGF^{AdKO}-HFD-AL and -IF mice. **(C)** Plasma alanine aminotransferase (ALT) activity. Values are mean ± SEM; two-tailed unpaired Student's *t*-test; **P* < 0.05 vs. VEGF^{AdKO}-HFD-AL. NS, not significant.
