# Supplementary material for: Intermittent fasting promotes adipose thermogenesis and metabolic homeostasis via VEGF-mediated alternative activation of macrophage
Source: Cell Res. 2017 Oct 17;27(11):1309–26. doi: 10.1038/cr.2017.126 (PMC5674160; doi:10.1038/cr.2017.126)
Supplement: Supplementary information, Figure S9 — Intermittent VEGF overexpression in BAT did not increase thermogenic activity in BAT. [file cr2017126x9.pdf]

Supplementary information, Figure S9

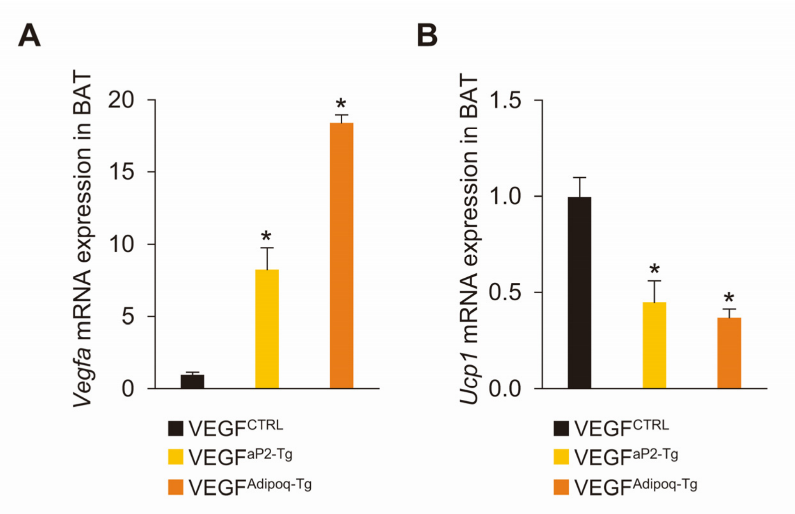

Figure S9 Intermittent VEGF overexpression in BAT did not increase thermogenic activity in BAT. (A) *Vegfa* mRNA expression in BAT of $VEGF^{aP2\text{-}Tg}$ and $VEGF^{Adipoq\text{-}Tg}$ mice showed successful VEGF overexpression in BAT. **(B)** *Ucp1* expression in BAT of $VEGF^{aP2\text{-}Tg}$ and $VEGF^{Adipoq\text{-}Tg}$ mice were significantly lower than control mice. Data are expressed as mean ± SEM ($VEGF^{CTRL}$: n = 10; $VEGF^{aP2\text{-}Tg}$: n = 6; and $VEGF^{Adipoq\text{-}Tg}$: n = 5); one or two-way ANOVA with Student-Newman-Keuls *post-hoc* analysis; *$P < 0.05$ vs. $VEGF^{CTRL}$.
