# Supplementary material for: Intermittent fasting promotes adipose thermogenesis and metabolic homeostasis via VEGF-mediated alternative activation of macrophage
Source: Cell Res. 2017 Oct 17;27(11):1309–26. doi: 10.1038/cr.2017.126 (PMC5674160; doi:10.1038/cr.2017.126)
Supplement: Supplementary information, Figure S10 — IF and adipose-VEGF overexpression induces alterative activation of macrophages. [file cr2017126x10.pdf]

Supplementary information, Figure S10

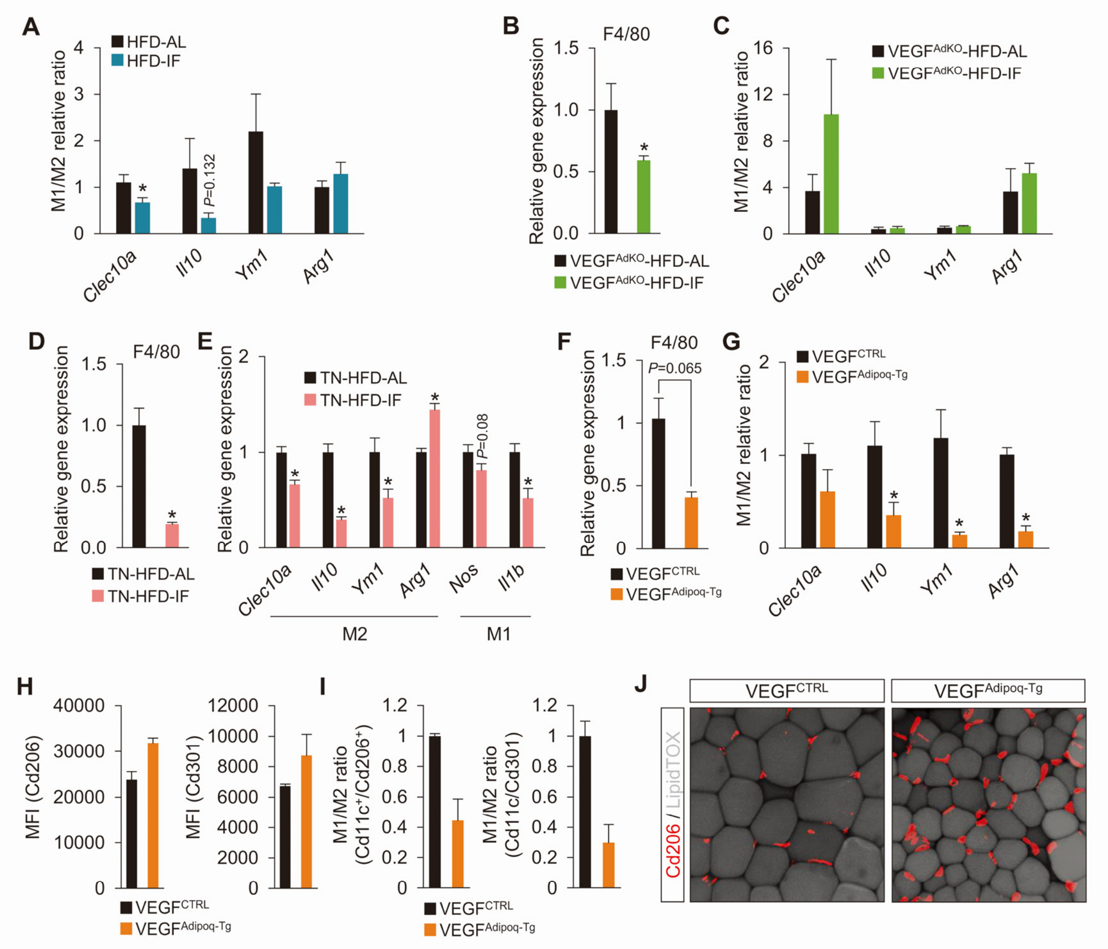

Figure S10 IF and adipose-VEGF overexpression induces alterative activation of macrophages. (A) M1/M2 macrophage ratio in HFD-AL and -IF mice. It was calculated by *Nos* gene expression as numerator and 4 different M2 markers as denominators. **(B)** Gene expression of pan-macrophage marker, F4/80 in VEGF^{AdKO}-HFD-AL and -IF mice. **(C)** M1/M2 macrophage ratio in VEGF^{AdKO}-HFD-AL and -IF mice. **(D)** F4/80 gene expression in TN-HFD-AL and -IF mice. **(E)** M1/M2 macrophage marker gene expression analysis in TN-HFD-AL and -IF mice. **(F)** F4/80 gene expression in VEGF$^{Adipoq\text{-}Tg}$ mice. **(G)** M1/M2 macrophage ratio in VEGF$^{Adipoq\text{-}Tg}$ mice. **(H)** Quantification of M2 macrophages in VEGF$^{Adipoq\text{-}Tg}$ mice using mean fluorescent

intensity (MFI) of Cd206- and Cd301-positive F4/80$^+$Cd11b$^+$ macrophages. **(I)** M1/M2 macrophage ratio in VEGF$^{Adipoq\text{-}Tg}$ mice using Cd11c$^+$/Cd206$^+$ and Cd11c$^+$/Cd301$^+$ macrophage number ratio. **(J)** Representative images of M2 marker Cd206-stained cells in whole-mount PWAT of VEGF$^{Adipoq\text{-}Tg}$ mice. Values are mean ± SEM; two-tailed unpaired Student's *t*-test; *$P < 0.05$ vs. HFD-AL, VEGF^{AdKO}-HFD-AL, TN-HFD-AL or VEGF^{CTRL} mice.
