# Supplementary material for: Intermittent fasting promotes adipose thermogenesis and metabolic homeostasis via VEGF-mediated alternative activation of macrophage
Source: Cell Res. 2017 Oct 17;27(11):1309–26. doi: 10.1038/cr.2017.126 (PMC5674160; doi:10.1038/cr.2017.126)
Supplement: Supplementary information, Figure S11 — 24-h fasting and acute adipose-VEGF induction lead to alterative activation of adipose macrophages. [file cr2017126x11.pdf]

Supplementary information, Figure S11

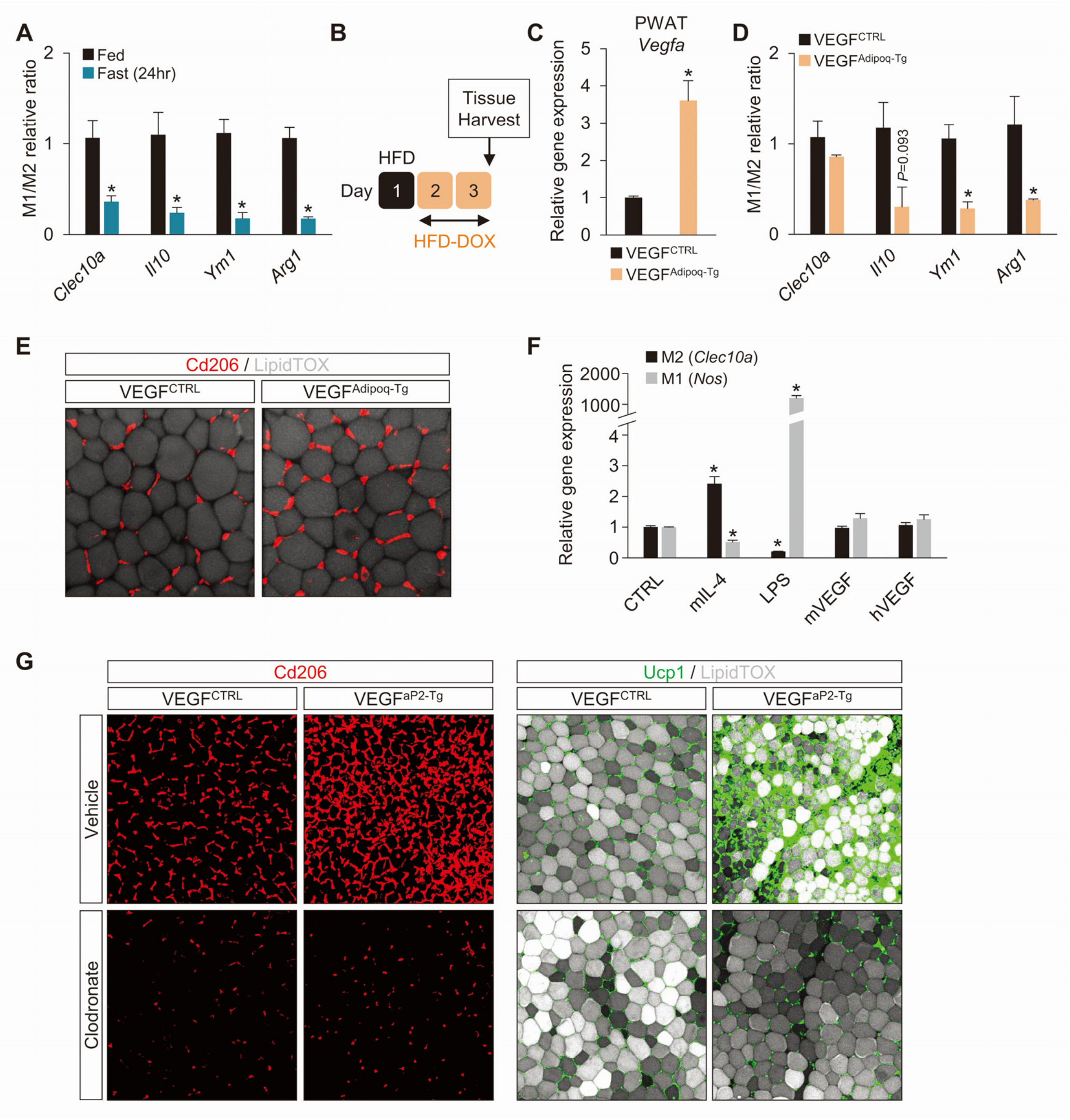

Figure S11 24-h fasting and acute adipose-VEGF induction lead to alterative activation of adipose macrophages. (A) M1/M2 macrophage ratio at feeding and 24 h fasting. **(B)** A schematic illustration of experimental schedule for acute adipose-VEGF induction by subjecting VEGF$^{Adipoq\text{-}Tg}$ mice to HFD-DOX diet for 48 h. **(C)** *Vegfa* mRNA expression in PWAT of VEGF$^{Adipoq\text{-}Tg}$ mice after acute adipose-VEGF overexpression. **(D)** M1/M2 macrophage ratio in VEGF$^{Adipoq\text{-}Tg}$ mice. **(E)**

Representative images of M2 marker Cd206-stained cells in whole-mount PWAT of VEGF$^{Adipoq\text{-}Tg}$ mice after acute induction. **(F)** M1 (*Nos*) and M2 (*Clec10a*) gene expression in Raw264.7 macrophage cells after treatments of mouse IL-4 (10 ng/mL), LPS (100 ng/mL), mouse VEGF (100 ng/mL) and human VEGF (50 ng/mL) for 12 h. **(G)** Representative images of M2 macrophages and Ucp1 expression in WAT after acute adipose-VEGF upregulation in VEGF$^{aP2\text{-}Tg}$ mice with treatments of vehicle or clodronate. *$P < 0.05$ vs. Fed, VEGF^{CTRL} mice or CTRL.
