# Supplementary material for: Intermittent fasting promotes adipose thermogenesis and metabolic homeostasis via VEGF-mediated alternative activation of macrophage
Source: Cell Res. 2017 Oct 17;27(11):1309–26. doi: 10.1038/cr.2017.126 (PMC5674160; doi:10.1038/cr.2017.126)
Supplement: Supplementary information, Figure S12 — VEGF expression in human WAT correlates with M2 macrophage and WAT browning. [file cr2017126x12.pdf]

Supplementary information, Figure S12

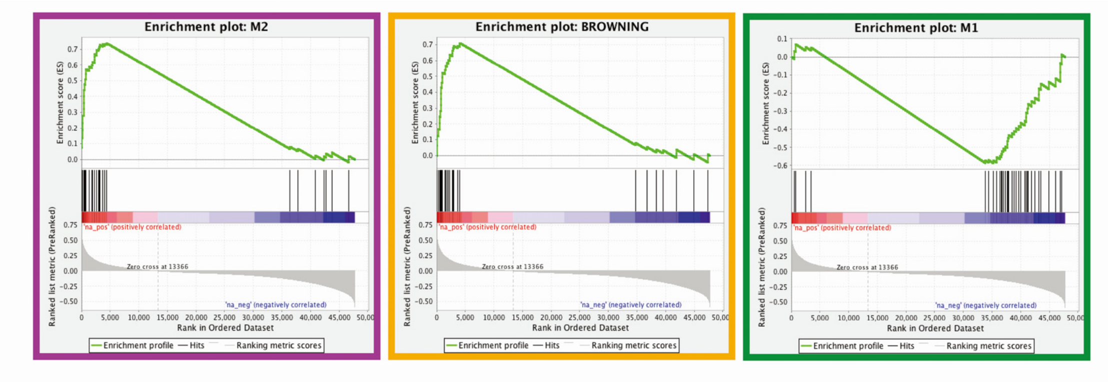

Figure S12 VEGF expression in human WAT correlates with M2 macrophage and WAT browning. Gene set enrichment plots show enrichment scores based on permutation tests of *VEGFA*-correlated M2, browning and M1 marker genes in human WAT.
