# Supplementary material for: Intermittent fasting promotes adipose thermogenesis and metabolic homeostasis via VEGF-mediated alternative activation of macrophage
Source: Cell Res. 2017 Oct 17;27(11):1309–26. doi: 10.1038/cr.2017.126 (PMC5674160; doi:10.1038/cr.2017.126)
Supplement: Supplementary information, Data S1 — Materials and Methods [file cr2017126x15.pdf]

Supplementary information, Data S1

Materials and Methods

Metabolic phenotyping

Body weight and food consumption were measured before and after fasting from 7 to 23 weeks of age. Body mass index (BMI) was calculated by dividing body weight by body length squared (body weight/body length^2). Caloric intake and feed efficiency (total energy intake/body weight gain, mg/Kcal) were calculated based on nutritional information (ND: 3.3 Kcal/g; HFD: 4.73 Kcal/g) provided by manufacturers. Body composition was analyzed using the EchoMRI-100 machine (Echo Medical Systems, Houston, TX, USA), which determines fat and lean mass in awake, non-anaesthetized mice.

For glucose and insulin tolerance tests, mice were subjected to intraperitoneal bolus injection of glucose (1 mg/g of body weight) or insulin (0.65 mU/g of body weight, Humulin®) after fasting overnight (14-16 h) or 6 h with water *ad libitum*, respectively. Blood glucose levels were measured at the indicated intervals using a glucometer (Contour NEXT, Bayer HealthCare). The homeostatic model assessment of insulin resistance (HOMA-IR) was calculated using values of fasting plasma glucose (FPG, mmol/L) and plasma insulin (PI, mU/L) as follows: HOMA-IR = FPG × PI /22.5.

Energy metabolism was evaluated through indirect calorimetry (Oxymax System, Columbus Instruments) over periods of 72 h. Oxygen consumption (VO_2) as an indicative of energy expenditure was normalized by body weight. In addition, to

adjust for body weight differences between AL and IF mice, individual VO_2 data were plotted in relation to body weight and analyzed using analysis of covariance (ANCOVA) [1, 2]. Locomotor activity and food intake were also measured simultaneously.

For histological analysis, harvested tissues were fixed in 4% paraformaldehyde and embedded in paraffin. Sections of 5 μm were stained with haematoxylin and eosin (H&E). The size of an adipocyte as cross-sectional surface area on randomly selected H&E stained sections was measured using Image J software.

Mouse plasma analyses were performed to measure adiponectin, insulin, leptin and resistin using MILLIPLEX® Multiplex Assays Using Luminex® (MADPNMAG-70K, MADKMAG-71K, and MAP2MAG-76K; EMD Millipore).

Transcriptome profiling by RNA-Seq

cDNA libraries were generated using the Nugen Ovation SPIA chemistry with a fully automated Wafergen's Apollo liquid handler, quantified by qPCR, normalized to equimolar concentrations, indexed, multiplexed (4 samples per flow cell), and subject to 1× 50-bp sequencing on the Illumina HiSeq-2000 sequencer to obtain an average of 47 million reads per sample with 97% of reads with the Q scores over 30. The reads were aligned to the GRCm38 mouse genome (Ensembl release 80) by *STAR* (v2.4.2a). Read alignments were merged and disambiguated, and a single BAM (Binary Alignment Mapped) file output per library or sample was used. BAM files were then

additionally filtered to remove reads with a mapping quality (MAPQ) less than 13, and all ribosomal and mitochondrial RNA reads. Alignments were assembled using Cufflinks (v2.2.1) using the –g parameter to construct a genome annotation file against the reference gene model (Ensembl release 80) and to identify novel transcripts. Raw read counts were obtained by mapping reads at the gene level using the Cufflinks assembled transcript annotation file with HTSeq-count tool from the Python package HTSeq, at http://www.huber.embl.de/users/anders/HTSeq/doc/count.html, using intersection-nonempty counting mode. EdgeR R-package (v3.12.1) [3] was then used to normalize the data, calculate transcript abundance (as counts per million reads (CPM)) and perform statistical analysis. Briefly, a common biological coefficient of variation (BCV) and dispersion (variance) was estimated based on a negative binomial distribution model. This estimated dispersion value was incorporated into the final EdgeR analysis for differential gene expression, and the generalized linear model (GLM) likelihood ratio test was used for statistics, as described in EdgeR user guide. Euclidean distance and complete linkage were used for hierarchical clustering of the samples using log2 transformed CPM (gene expression) values. Euclidean distance and Ward's minimum variance method of clustering were used to obtain the gene clusters used Z-score normalized gene expression values. All statistical analyses and data visualization were done in *R* using *R* basic functions and the following packages: clusterProfiler (v2.4.3), gplots (v3.0.1), and stats (v3.2.2). Raw RNA-seq data is available upon request.

Human adipose gene correlation analysis

RNA-Seq data of human adipose tissue samples (The GTEx Analysis V6p) were downloaded from the Genotype-Tissue Expression (GTEx) Project portal website (www.gtexportal.org) [4], and the RPKM (reads per kilobases and a million reads) values were normalized to the Z scores by gene across the samples. The lists of genes specifically associated with M1/M2 macrophages or beige/brown adipocytes were assembled by database and literature search (Supplementary information, Table S2), and the genes were hierarchically clustered by a correlation-based average linkage and visualized as a heat map by using the *Biopython* and the *Resportlab* Python packages. Pearson's correlation coefficients between expression levels of *VEGFA* and every gene (n = 47,757) in the dataset were calculated, and the genes were ranked by the coefficients (positive to negative). Statistical significance of correlation was obtained by using the *Scipy.stats.pearsonr* function in Python, and the *P* values were adjusted for multiple testing by the Benjamini-Hochberg method by using the *Stats.correct_pvalues_for_multiple_testing* function. To test if M1/M2 macrophage- and beige/brown adipocyte--associated genes were enriched among genes that were either positive or negatively correlated with *VEGFA* expression, we performed a rank-based gene set enrichment analysis (GSEA) on each gene set by including only the genes with adjusted *P* values < 0.05) with the *GSEAPreranked* software with default parameters (software.broadinstitute.org/gsea) [5].

Gene expression analysis by qPCR

Total RNA was extracted from tissues using RNeasy Lipid Tissue Kit (Qiagen), and complementary DNA was synthesized from 2 μg of RNA using M-MLV reverse transcriptase (Invitrogen) with oligo(dT). Gene expression assay was conducted using SYBR Green methods on Viia7 (Applied Biosystems), and relative cycle threshold (CT) values were normalized by *36b4* or *Tbp*. Primers sequences are available upon request.

Whole-mount adipose tissue Imaging

Mice were subjected to perfusion with 1% paraformaldehyde in PBS via cardiac puncture, then adipose tissues were harvested and fixed with 1% paraformaldehyde for 1 h at room temperature. For *Ucp1-Cre*;*Rosa26*$^{mT/mG}$ mice, adipose tissues were washed with PBS three times and subjected to image acquisition using a confocal laser scanning microscope (Zeiss LSM).

For whole-mount adipose blood vessel visualization and M2 macrophage staining, perigonadal white-adipose tissues were harvested and fixed with 1% paraformaldehyde in PBS, followed by incubation for 1 h at room temperature with 0.3% Triton X-100 in PBS (PBST) solution containing 5% goat serum (Jackson ImmunoResearch). After blocking, the samples were incubated overnight at 4 °C with following primary antibodies: anti-PECAM-1 (Millipore, #MAB1398Z, hamster monoclonal clone 2H8, 1:200), anti-perilipin (Fitzgerald Industries International, #20R-PP004, guinea pig polyclonal, 1:200), anti-Cd206 (clone MR6F3, eBioscience, 1:500) or anti-Ucp1 (Abcam, #Ab23841, Rabbit polyclonal, 1:1000). After several

washes in PBST, tissues were incubated for 2 h at room temperature with the following secondary antibodies: goat anti-hamster Alexa Fluor® 594 (Jackson ImmunoResearch, #107-585-142, 1:500), goat anti-guinea pig Alexa Fluor® 488 (Jackson ImmunoResearch, #106-545-003, 1:500) and goat anti-rabbit Alexa Fluor® 488 (Jackson ImmunoResearch, #111-545-144, 1:500). For whole-mount staining of mature adipocytes, LipidTOX™ Deep Red Neutral Lipid Stain (ThermoFisher; #H34477; 1:1000) was used. Fluorescent signals were visualized and digital images were obtained by using a Nikon A1R confocal microscope system. Blood vessel densities were made by photographic analysis using the ImageJ software (http://rsb.info.nih.gov/ij). PECAM$^+$ area were measured and presented as percentages per the measured.

Flow cytometry

The visceral white adipose tissue was finely minced and were incubated for 30-45 min of digestion at 37 °C with tissue digestion cocktail containing 1 mg/mL collagenase type II (Worthington Biochemical Corporation) in DMEM/F12 media, with 1% fatty acid-free BSA and 100 U/mL DNase I. The tissue digest was neutralized with serum medium (DMEM/F12 media with 10% FBS), then was filtered through a 200 μm cell strainer. The floater fraction was discarded and red blood cell lysis buffer (Sigma-Aldrich) was added to the stromal vascular fraction. The cells were resuspended in FACS buffer (1× HBSS with 1 mM EDTA, 25 mM HEPES, 1% FBS) and was blocked with rat anti-mouse CD16/CD32 (1:100, clone 2.4G2, BD

Pharminogen™) for 10 min. To observe macrophage populations in the SVF, the cells were stained with rat anti-mouse CD45 (1:100, clone 30-F11, eBioscience), rat anti-mouse/human CD11b (1:200, clone M1/70, BioLegend), rat anti-mouse F4/80 (1:50, clone BM8, eBioscience), Armenian hamster anti-mouse CD11c (1:100, clone N418, BD Biosciences), rat anti-mouse CD206 (1:200, clone MR6F3, eBioscience), rat anti-mouse CD301 (1:100, clone LOM-14, BioLegend), and fixable viability dye (1:1000, REF 65-0865, eBioscience) for 30 min at 4 °C. The dead cells were gated out, then pan macrophages were identified as $CD45^{+}CD11b^{+}F4/80^{+}$. M1 macrophages were further gated with CD11c, and M2 macrophages were gated using CD206 and CD301, as previously shown [6]. Data were acquired using GALLIOS from Beckman and analyzed with FlowJo.

Depletion of adipose macrophages and β-adrenergic receptor block

To deplete macrophage in adipose tissues, mice were subjected to injection of liposomal clodronate (Cldorosome, Encapsula) according to the manufacturer's instruction. Briefly, Clodorosome was administrated at 50 mg/kg through intraperitoneal injection for 2 consecutive days. On Day 3 after Clodrosome injection, the mice were fasted for 24 h or subjected to 45% HFD containing doxycycline for 48 h, then PWAT was collected for further analysis. Depletion of macrophages were verified by qPCR-based F4/80 gene expression as well as whole-mount immunostaining.

To block sympathetic activation by fasting, two different β-adrenergic receptor

(AR) antagonists, SR-59230A (β_3-AR specific; 5 mg/kg i.p.) and Propranolol (non-specific; 5 mg/kg i.p.) were injected two times (at 0 and 12 h) during 24-h fasting period.

Macrophage RAW264.7 cell line culture

Mouse macrophage RAW264.7 cell line (ATCC® TIB-71™) was cultured in DMEM supplemented with 10% fetal bovine serum containing penicillin-streptomycin at 37 °C with 5% CO_2. The cells were cultured at density of 1×10^6 until they nearly reached confluency. When confluent, the cells were starved with 2% FBS in DMEM for 3 h, then treated for 12 h as follows: 10% and 2% serum as negative control, 10 ng/mL of mouse IL-4 (Peprotech) and 100 ng/mL of LPS (Sigma) as positive controls to induce M2 and M1 polarization, respectively, 100 ng/mL recombinant mouse VEGF 164 protein (R&D), and 50 ng/mL recombinant human VEGF 165 protein (R&D). The cells were collected for gene expression analysis.

SUPPLEMENTAL REFERENCES

1 Speakman JR, Fletcher Q, Vaanholt L. The '39 steps': an algorithm for performing statistical analysis of data on energy intake and expenditure. *Dis Model Mech* 2013; **6**:293-301.

2 Tschop MH, Speakman JR, Arch JR *et al.* A guide to analysis of mouse energy metabolism. *Nat Methods* 2012; **9**:57-63.

3 Robinson MD, McCarthy DJ, Smyth GK. edgeR: a Bioconductor package for differential expression analysis of digital gene expression data. *Bioinformatics* 2010; **26**:139-140.

4 Consortium GT. Human genomics. The Genotype-Tissue Expression (GTEx) pilot analysis: multitissue gene regulation in humans. *Science* 2015; **348**:648-660.

5 Subramanian A, Tamayo P, Mootha VK *et al.* Gene set enrichment analysis: a knowledge-based approach for interpreting genome-wide expression profiles. *Proceedings of the National Academy of Sciences of the United States of America* 2005; **102**:15545-15550.

6 Cho KW, Morris DL, Lumeng CN. Flow cytometry analyses of adipose tissue macrophages. *Methods Enzymol* 2014; **537**:297-314.
